# Multi-postpolymerization Functionalization of PLA Enabled by Sulfide Tetrazines as a Single End-Group Moiety

**DOI:** 10.1021/acspolymersau.5c00045

**Published:** 2025-07-17

**Authors:** Jesper S. Willems, Dulce M. Sánchez-Cerrillo, Katerina Gavriel, Kevin Neumann

**Affiliations:** Systems Chemistry Department, Institute for Molecules and Materials, Radboud University Nijmegen, Heyendaalseweg 135, 6525 AJ Nijmegen, The Netherlands

**Keywords:** ROP, PEGylation, bioorthogonal chemistry, tetrazine, nanomedicine, postpolymerization functionalization

## Abstract

The synthesis of well-defined and multifunctional polymeric systems for drug delivery is a topic of intense research. However, a major limitation in the functionalization of these systems for nanomedical applications is the reliance on toxic catalysts or non-biocompatible strategies for polymer coupling and ligation, as well as the need for extensive purification steps when multiple functionalization reactions are performed. In this work, we introduce tetrazines containing both a methyl sulfide and hydroxyl functionality as the initiator for ring opening polymerization of l-lactide. We demonstrate that the obtained polymer can be successfully conjugated to mPEG-SH by chemoselective tetrazine-thiol exchange in a traceless manner. The byproduct, methanethiol, is easily eliminated during the course of the reaction, thus avoiding the use of a toxic catalyst. Additionally, the same end group allows for further functionalization via inverse electron demand Diels–Alder chemistry using *trans*-cyclooctene. These findings highlight the potential of TeTEx to access complex polymeric systems for biomedical applications by enabling two distinct postpolymerization functionalizations at a single end group, notably in a truly traceless manner.

## Graphical abstract

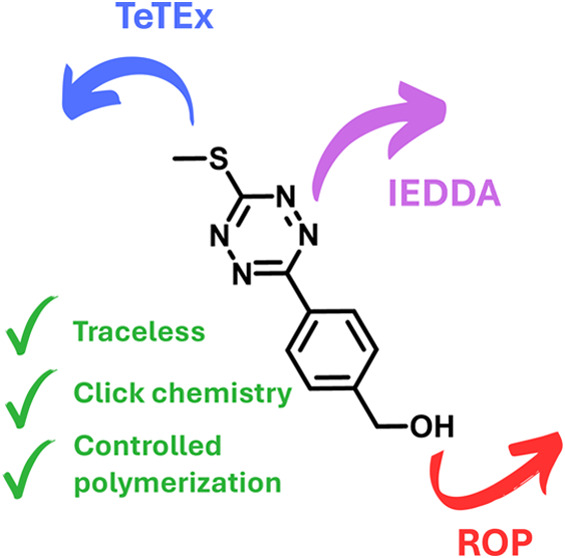

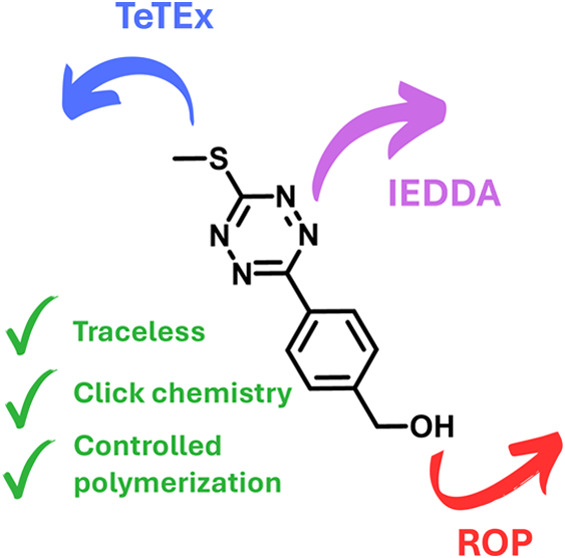

## Introduction

Polyesters, such as poly­(lactide) (PLA), have been widely used for applications in nanomedicine. Due to the biocompatibility of PLA, several formulations including either the homopolymer or the copolymer poly­(lactide-*co*-glycolide) (PLGA) have been approved by the Food Drug Administration and the European Medicine Agency.^1–3^ Consequently, the development of nanocarriers containing PLA with advanced features such as polyethylene glycol (PEG) as a hydrophilic segment to increase the stealth character is a topic of intense research.^4,5^

PLA can be synthesized using different routes including the polycondensation of lactic acid or the ring opening polymerization (ROP) of enantiomeric pure forms of lactide, namely, l-lactide, d-lactide, or l,d-lactide.^6,7^ In the case of the polycondensation of lactic acid, being a non-controlled polymerization, one of the main limitations is the production of polymeric chains with broad dispersity. On the other hand, ROP of lactides enables a controlled polymerization with typically narrow dispersity polymers. This characteristic is crucial for tailoring the properties and applications of this material in the field of nanomedicine.^8^ The synthesis of PLA by ROP is often preferred to obtain polymers with high molar mass and narrow dispersity (*D̵*). The polymerization can be carried out utilizing heavy metals as catalysts including tin as one of the most common catalysts used for such purpose.^9–11^ However, these metals represent a risk in terms of medical applications due to their cytotoxicity.^12,13^ During the past decade, amidine bases, considered safe for biomedical applications, have emerged as alternative catalysts for ROP. Examples of such bases include but are not limited to 1,8-diazabicyclo[5.4.0]­undec-7ene (DBU), 1,5,7-triazabicyclo[4.4.0]­dec-5-ene (TBD), and 7-methyl-[1,5,7-triazabicyclo[4.4.0]­dec-5-ene)] (mTBD). Their mechanism includes the activation of the initiator or both, initiator and monomer.^14–16^

A major challenge remaining is the efficient functionalization of PLA to construct well-defined and complex macromolecular architectures for nanomedicinal applications. In particular, conjugation to PEG for the construction of amphiphilic block copolymers with stealth characteristics, as well as conjugation to active cargos, such as fluorophores and drug molecules, is of high interest. Such functionalization can be carried out using different techniques including chemical conjugation of PEG to linear PLA chains, or by the direct polymerization of lactide utilizing the end-functionalized hydroxyl group of PEG as a macroinitiator.^17–20^ However, one must be careful that the remaining catalysts or coupling agents do not cause toxicity. For this reason, it is essential to establish so-called traceless functionalization procedures with non-toxic side products or purification steps that enable high yields, but yet maintaining their biocompatibility. In addition, for construction of well-defined polymeric systems, multiple functionalization is often beneficial, yet such approaches often require extensive purification steps alongside significant synthetic overheads. Previously, our group reported the so-called tetrazine-thiol exchange (TeTEx) which allows the chemoselective functionalization of complex small molecules by reversible click chemistry using unsymmetrical sulfide tetrazines.

Notably, these reversible systems can also be locked in a bioorthogonal manner by the addition of a dienophile. Both mechanisms provide high yields and due to their mild reaction conditions are suitable for nanomedical applications.^21^ The dynamic nucleophilic substitution of tetrazines have been also investigated for the fabrication of polymeric networks.^22,23^ Additionally, tetrazines have been employed as initiators for ROP of lactones or *N*-carboxyanhydride (NCA) with subsequent functionalization by inverse electron demand Diels–Alder (IEDDA) or strain-promoted alkyne–azide cycloaddition (SPAAC).^24,25^ However, to the best of our knowledge, systems with the possibility of multiple functionalizations from the same functional group have not been investigated. Therefore, here, we introduce the ROP of l-lactide initiated by a disubstituted tetrazine containing a methyl sulfide moiety that acts as a click handle and a benzyl alcohol that initiates the ROP of l-lactide. We demonstrate the utility of TeTEx to provide access to a block copolymer, specifically as a PEGylation technique for PLA without the use of toxic catalysts, in quantitative yields (Figure 1a). Furthermore, we demonstrated that the tetrazine moiety positioned between the two polymeric segments can undergo an IEDDA reaction with *trans*-cyclooctene (TCO) (Figure 1b), thereby offering a strategy that enables multiple traceless functionalization steps from a single residue. We envision that our method opens the possibility for the synthesis of complex polymeric structures for nanomedical applications.

**Figure 1:**
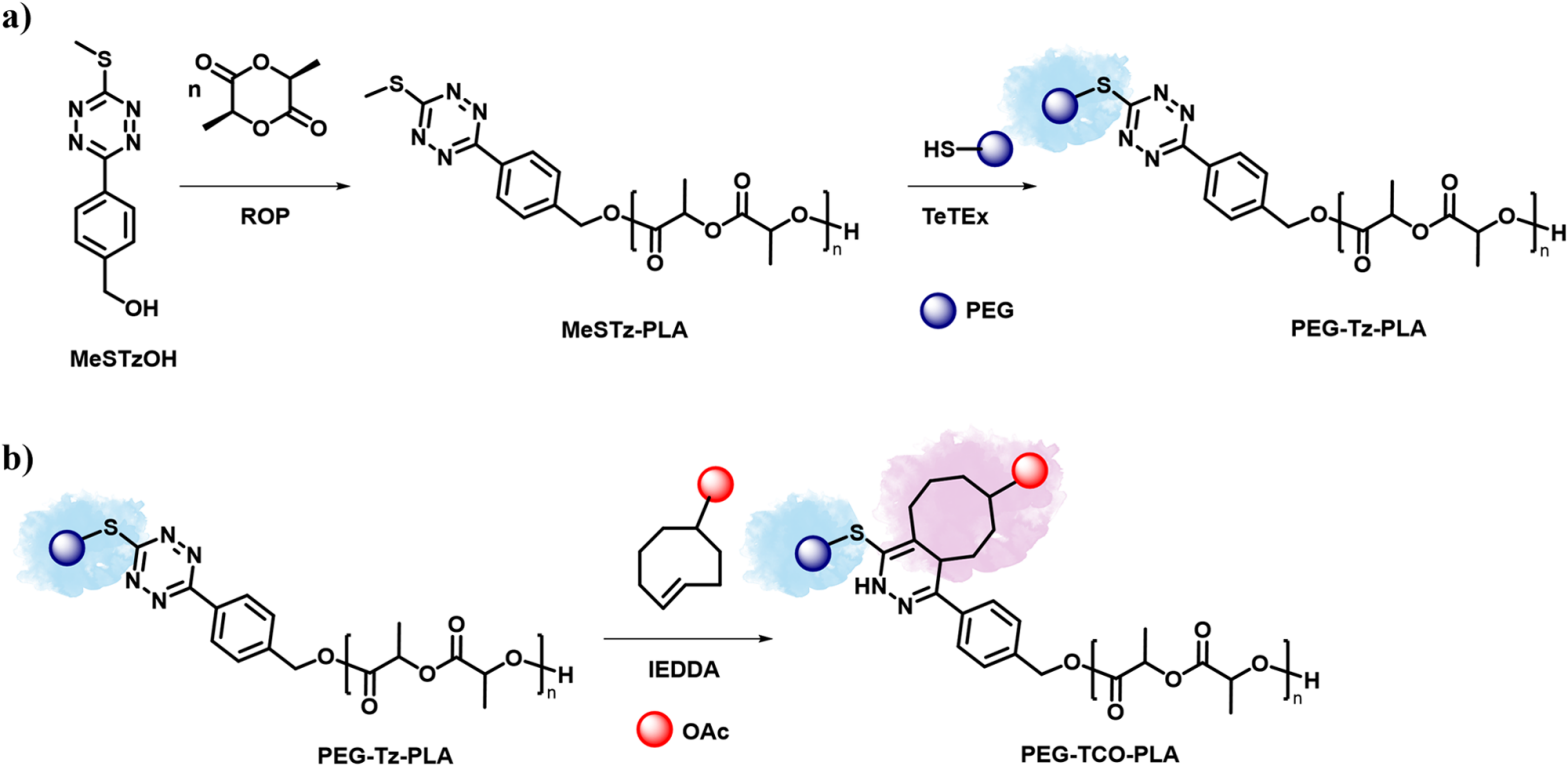
(a) Schematic representation of the synthesis of PLA utilizing MeSTzOH as the initiator and subsequent postpolymerization functionalization with PEG-SH via TeTEx. (b) Second traceless postpolymerization functionalization between PEG-Tz-PLA via bioorthogonal IEDDA between tetrazine and TCO.

## Results and Discussion

### ROP of l-Lactide Utilizing MeSTzOH as the Initiator

In order to assess suitable conditions for ROP of l-lactide utilizing methyl-sulfide tetrazine with a benzyl alcohol moiety (MeSTzOH) as the initiator, a series of experiments were conducted. Since previous studies have shown that tetrazines may degrade more rapidly in the presence of organocatalysts such as DBU or TBD,^24,26^ our initial experiments aimed to demonstrate that polymerization can proceed at 1.0 M in THF using mTBD as an organocatalyst (Figure 2a).

**Figure 2:**
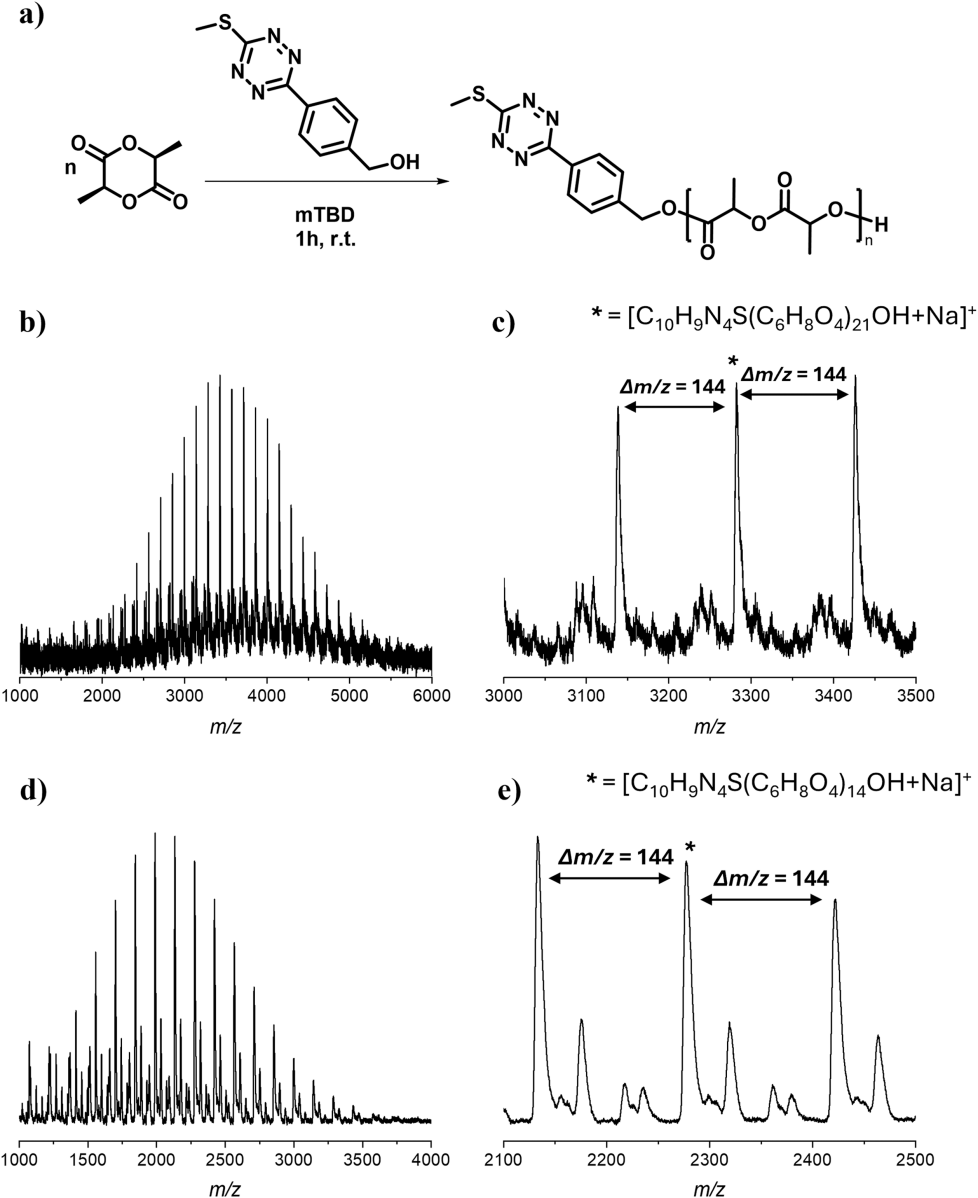
(a) Schematic representation of the ring-opening polymerization of l-lactide initiated by MeSTzOH. (b) MALDI-ToF mass spectrum of Tz-PLA from the kinetic study in THF at 5 min (c) Zoom in to the most abundant *m*/*z* region. (d) MALDI-ToF mass spectrum of Tz-PLA from the kinetic study in DCM at 5 min (e) Zoom in to the most abundant *m*/*z* region.

MALDI analysis (Figure 2b,c) of reaction samples taken after 5 min reaction time confirmed that the main distribution corresponds to PLA chains containing MeSTzO- as the end functional group. The mass spectrum from the sample at 5 min in DCM further confirmed that the polymerization of l-lactide is successfully initiated by MeSTzOH (Figure 2d,e) regardless of the polarity of the solvent.

Once we observed that polymerization of l-lactide can be initiated by MeSTzOH, we evaluated the effect of the molarity at 1.0, 0.8, and 0.2 M utilizing THF as a solvent. Although higher conversions were observed for 0.8 and 1.0 M, a shoulder in the GPC elugrams was observed after 30 min of reaction time (Figure S1). This shoulder was likely caused by transesterification interactions, which have been reported for high conversions during ROP of l-lactide when utilizing active organocatalysts.^27–29^ After 3 h reaction time, polymerization carried out at 0.8 M resulted in a molecular weight *M*_n_ = 14.64 kDa and dispersity *D̵* = 1.21 compared to *M*_n_ = 18.12 kDa, and *D̵* = 1.15 for the polymerization conducted at 1.0 M. Notably, good control in the course of the polymerization was observed at 0.2 M, even after prolonged time (23.5 h, 87%, conv., *D̵* = 1.18). However, due to the longer reaction times required and the chain transfer interactions, we decided thento evaluate the effect of solvent at 0.3 M utilizing less polar solvents compared to THF. Higher conversion and relatively low dispersity were observed when selecting toluene as a solvent. Notably, a less pronounced shoulder was observed after 30 min reaction time with monomer conversion of 70% determined by ^1^H NMR analysis and *D̵* = 1.15 (Figure 3a).

**Figure 3:**
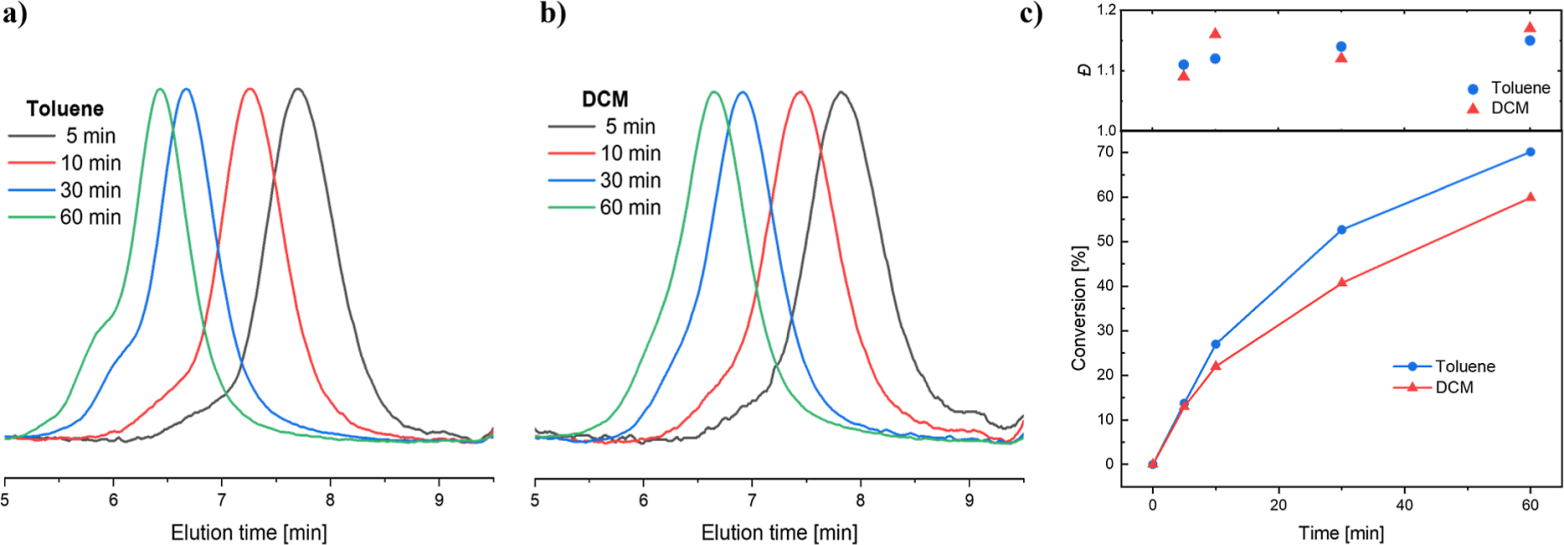
(a) Overlay of GPC elugrams for the kinetic study of the ROP of l-lactide initiated by MeSTzOH in Toluene at 0.3 M. (b) Overlay of GPC elugrams for the kinetic study of the ROP of l-lactide initiated by MeSTzOH in DCM at 0.3 M. (c) Evolution of conversion and dispersity (*D̵*) as a function of time. GPC elugrams measured in DMAC with RI detection.

In contrast, when utilizing DCM as a solvent, after 60 min, 60% conversion was observed with an unimodal distribution and *D̵* = 1.17 (Figure 3c). Hence, we concluded that DCM and a concentration of 0.3 M provides suitable conditions for the upscale polymerization of l-lactide utilizing MeSTzOH as the initiator compared to THF or Toluene (Figure 3b). Overall, relatively high conversion was reached after 60 min of reaction time, and low *D̵* was maintained during the course of the polymerization (Figure 3c). Purification of the polymer was carried out by precipitation in cold methanol yielding a pink polymer powder (*M*_n_ = 10.96 kDa, *D̵* = 1.15). This characteristic pink color is attributed to the tetrazine end group due to the *n* → π* transitions in the visible spectrum.^30,31^

### Synthesis of PEG-Tz-PLA by TeTEx with α-Methoxy-ω-Mercapto-PEG

With the PLA bearing a MeSTz as end group in hand, we turned our attention to establishing a robust method that allows the conjugation of thiol containing polymers to the sulfide tetrazine bearing terminus by means of TeTEx. As a proof of concept, we employed first α-methoxy-ω-mercapto-PEG (PEG-SH) (*M*_n_ = 2 kDa) for such chemistries. Previously, our group reported this mechanism with the use of different asymmetrical methyl sulfide tetrazines and thiol-containing molecules. The synthesis of cyclic peptides with these two moieties as end-functional groups in a peptide was also achieved.^32^ Different conditions were evaluated for the polymer conjugation and are summarized in Table S1. Due to the amphiphilic character of the obtained polymer, the most suitable TeTEx conditions involved reactions of MeSTz-PLA with PEG-SH and triethylamine in an organic solvent, namely, acetonitrile. Phase separation was observed utilizing dioxane and 10xPBS (Figure S2), while for DMSO and 10xPBS, no conjugation was observed. This is in contrast to previously reported conditions by the Fox group and us and highlights the challenge to establish lcick chemistries for polymer modifications.^21,33^

The product was obtained within an hour of reaction time. It is important to mention that by using TeTEx, the byproduct formed is MeSH which is easily eliminated by saturation of the reaction with an inert gas during the conjugation. The traceless character offers many advantages including easy purification of the products and, most importantly, the absence of toxic catalysts. Therefore, this mechanism offers new possibilities for the easy coupling of polymers for biomedical applications. By DOSY analysis, we observed a single diffusion coefficient for the PEG signal at δ = 3.67 ppm and the signals at 5.19 and 1.61 ppm, corresponding to the methene and methylene protons of PLA, respectively (Figure 4a). Importantly, diffusion signals corresponding to free PEG-SH were not observed. In Figure 4b, GPC analysis confirmed the successful PEG-Tz-PLA conjugation, observing a shift to higher molecular weight, maintaining a narrow dispersity (*M*_n_ = 12.36 kDa, *D̵* = 1.11). It is important to note that by ^1^H NMR analysis, no change of the aromatic signals at δ = 8.58–7.53 was observed, corresponding to the benzyl moiety of the tetrazine, indicating that the tetrazine between the two polymers remained stable after TeTEx (Figure 4c). FT-IR analysis further confirmed the presence of the tetrazine in the block copolymer (Figure S3, S4).

**Figure 4:**
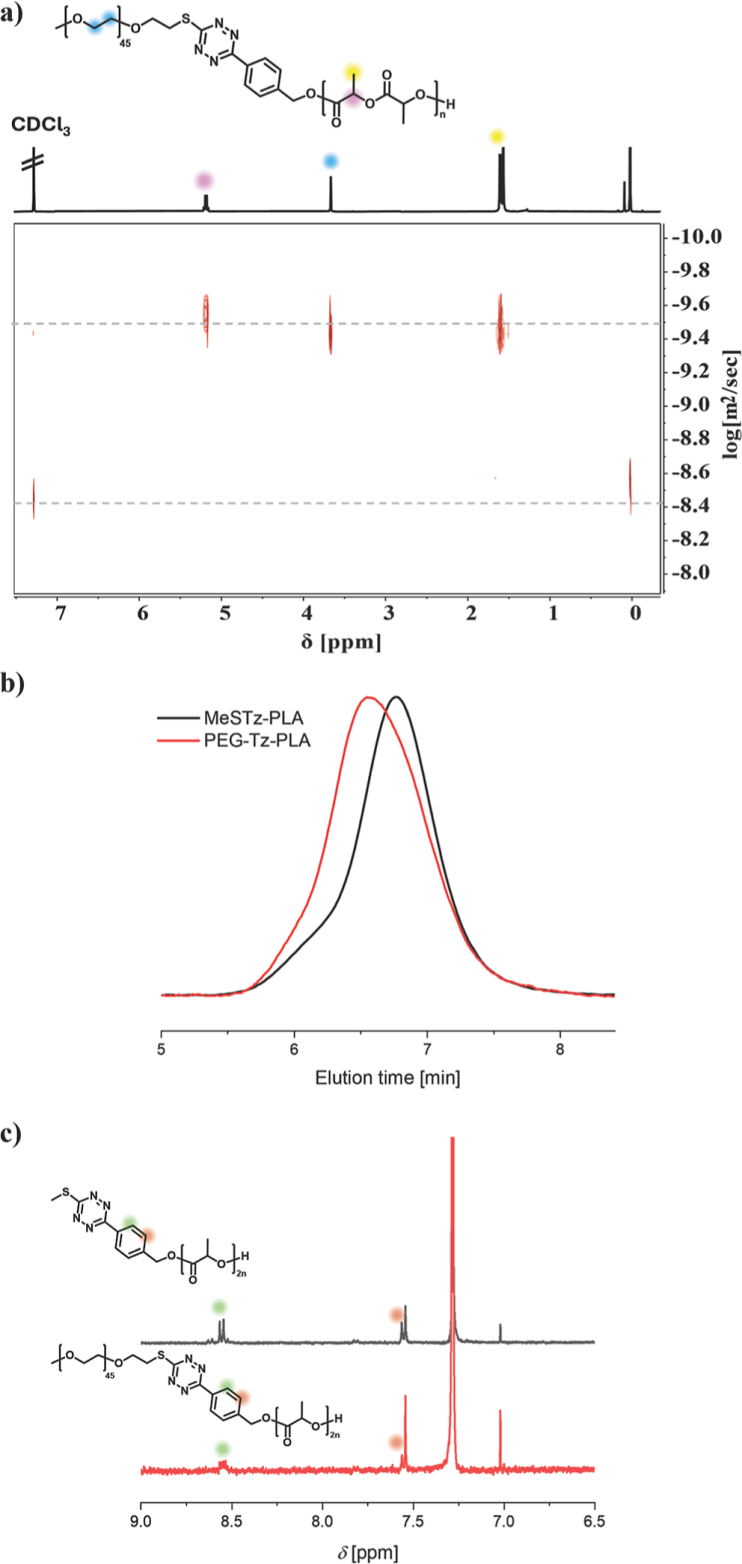
(a) DOSY NMR spectrum (500 MHz, CDCl_3_) of PEG-Tz-PLA. (b) Overlay of GPC elugrams (DMAc, RI detection) of MeSTz-PLA and PEG-Tz-PLA. (c) ^1^H NMR spectra overlay (400 MHz, CDCl_3_) of MeSTz-PLA and PEG-Tz-PLA.

Using the same protocol, PEG_4_-Tz-PLA and PEG_5kDa_-Tz-PLA were obtained, featuring significantly shorter and longer PEG chains (*M*_n_ = 224 g mol^–1^ and *M*_n_ = 5 kDa), respectively. These results demonstrate that the technique is suitable for the traceless conjugation of PEG–PLA constructs bearing a tetrazine moiety between the two polymer blocks, regardless of the PEG molecular weight (Figures S5 and S6).

### Reversibility of TeTEx by Addition of GSH

Previously, the reversibility of TeTEx was proved by the addition of an excess of l-glutathione.^21^ However, under various conditions, in our system, such reversibility was not observed for PEG_2kDa_-Tz-PLA, even after a prolonged time of 24 h (Figure 5a). We speculated that the reversibility is somewhat restricted due to steric effects caused by the polymeric chains around the tetrazine that limit the reactivity of the sulfide present in the tetrazine. This hypothesis was supported by a pronounced molecular weight shift observed upon TeTEx reversal of PEG_4_-Tz-PLA, displaying a shorter PEG residue (Figure 5b). Further studies are required to explore this effect by conjugation of MeSTz-PLA with other smaller molecules containing a thiol group.

**Figure 5:**
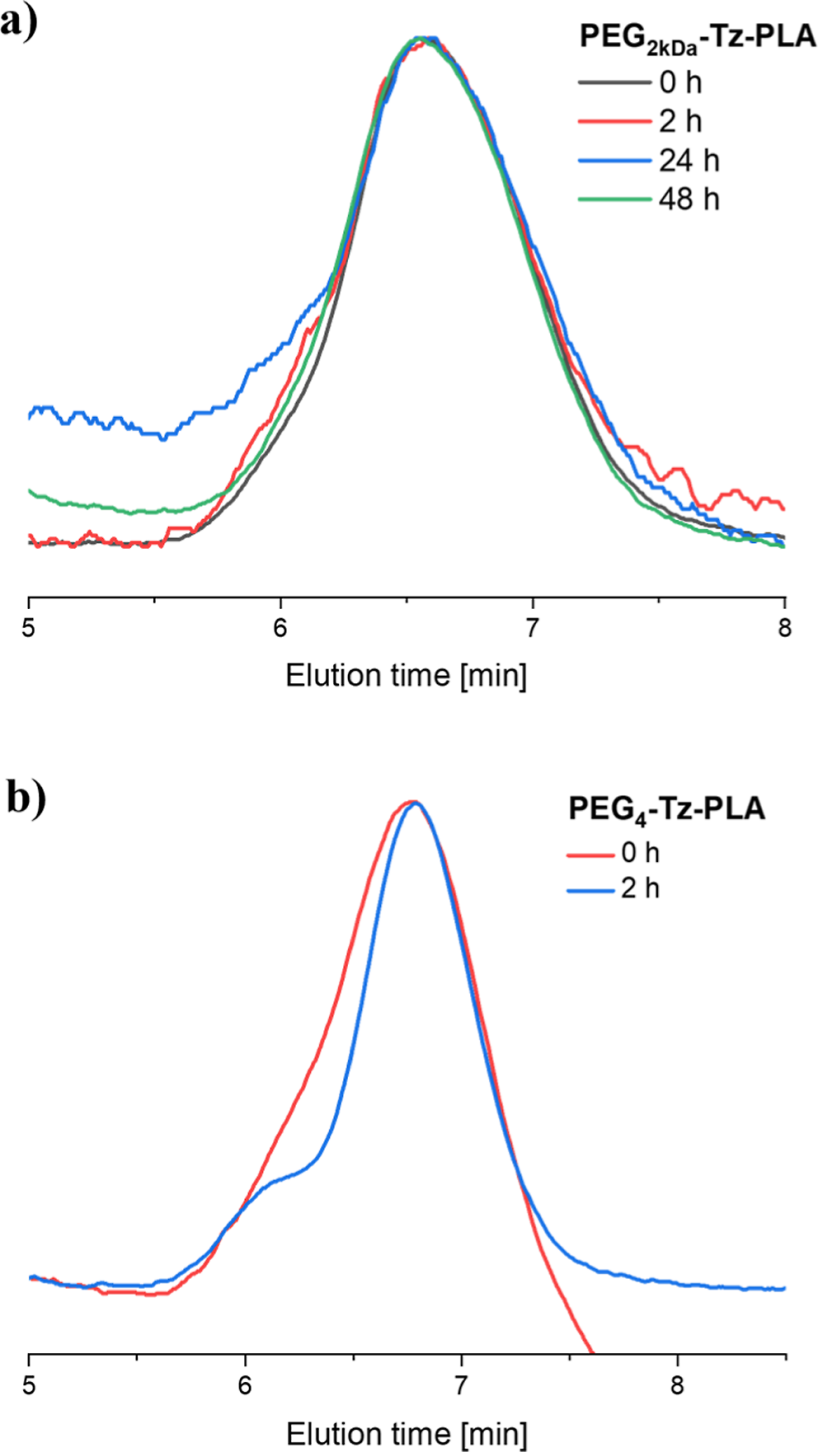
Overlay of GPC elugrams (DMAc, RI detection) for testing the reversibility of TeTEx upon addition of an excess of l-glutathione in ACN for (a) PEG_2kDa_-Tz-PLA and (b) PEG_4_-Tz-PLA.

### Bioorthogonal IEDDA of TCO with PEG-Tz-PLA

Bioorthogonal reactions of tetrazines by IEDDA chemistries have been extensively investigated and allows for the synthesis of complex molecules for biological applications as well as prodrug activation strategies.^26,34–37^ Having demonstrated that TeTEx enables the synthesis of a defined block copolymer containing a tetrazine in between these two segments, we sought to further investigate its functionalization by the IEDDA reaction using TCO. Thus, PEG_2kDa_-Tz-PLA block copolymer was reacted with TCO for 30 min. The reaction mixture turned colorless, which was the first indication of quantitative ligation. In addition, ^1^H NMR revealed a shift of the aromatic signals at δ = 8.58–7.53 ppm from the benzyl moiety in the tetrazine to the lower field (Figure 6a), confirming the effectiveness of the reaction. Notably, a minor shift to a higher molecular weight was observed in Figure 6b by GPC analysis. Further confirmation of the successful functionalization was demonstrated by UV–vis analysis (Figure S7), in which the maximum at around 536 nm vanished after the reaction with TCO, indicating the conversion to a dihydropyridazine.^38^ These results indicate that although the reversibility of TeTEx is sterically limited by the polymeric chains, the tetrazine moiety retains reactivity toward IEDDA. Our findings demonstrate that double functionalization is achieved by a single end group functionality under mild conditions. These results indicate that the use of asymmetrical tetrazines containing methyl sulfide and an alcohol is a promising approach for the synthesis of complex polymeric systems with nanomedical applications.

**Figure 6:**
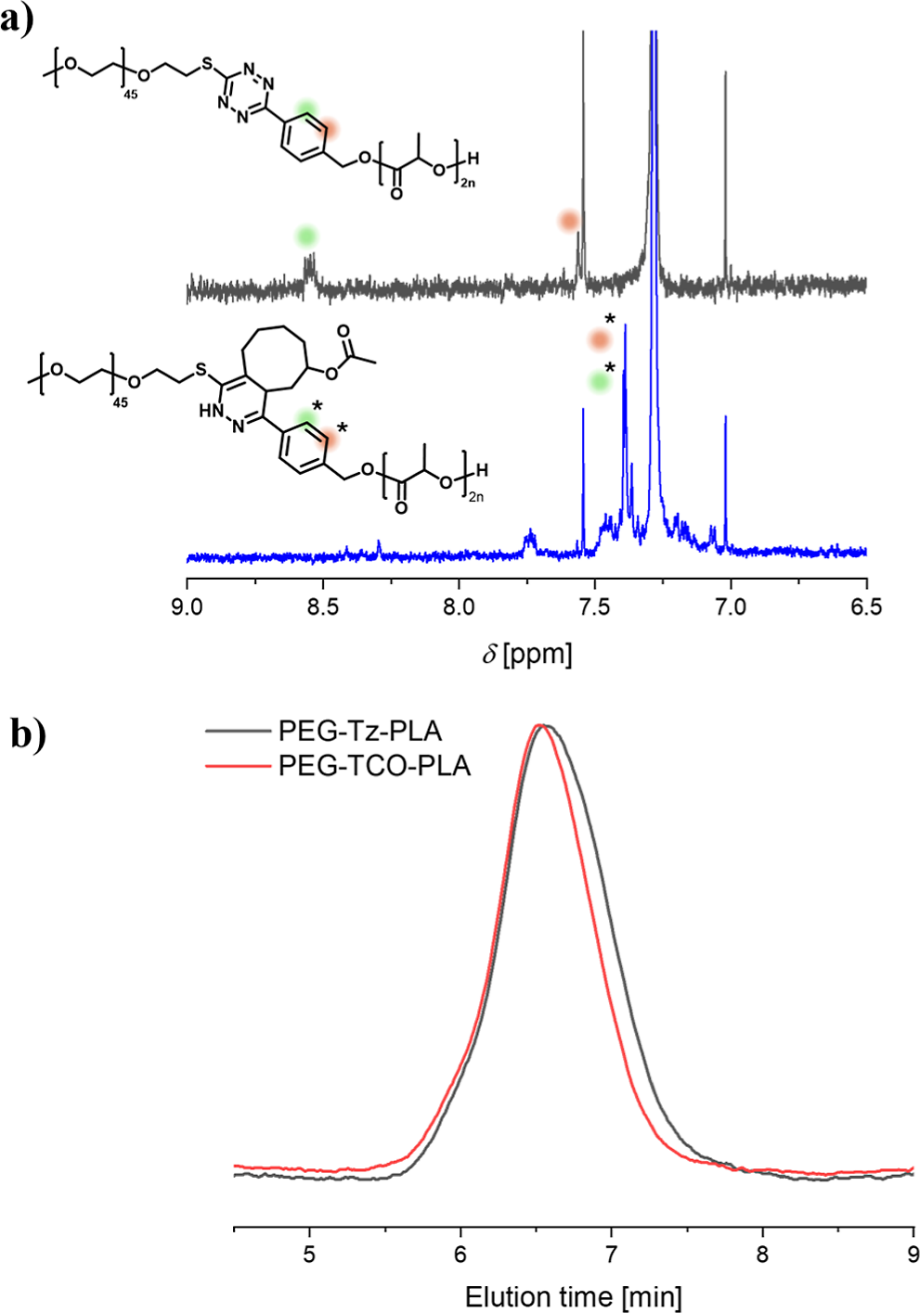
(a) ^1^H NMR spectra overlay (400 MHz, CDCl_3_) of PEG-Tz-PLA and PEG-TCO-PLA. (b) Overlay of GPC elugrams (DMAc, RI detection) of PEG-Tz-PLA and PEG-TCO-PLA.

## Conclusion

In summary, we showed for the first time that asymmetrical tetrazines bearing a methyl sulfide moiety can be used as initiators for the synthesis of PLA, resulting in a defined sulfide tetrazine end group. This feature allows multiple postpolymerization functionalizations via TeTEx and subsequent IEDDA chemistry. The click-character of both functionalizations entails quantitative conversions under mild conditions. We demonstrated the utility of this approach by accessing an amphiphilic block copolymer using PEG-SH. Additionally, we introduced the ligation with TCO in the polymeric chain by IEDDA. The synthesis of polymers with the possibility of multiple functionalization in one molecule opens new synthetic routes for the construction of complex macromolecular systems for nanomedical applications. Notably, we foresee that the reactivity toward IEDDA enables implementation of click-to-release chemistries in the future. Although TeTEx is in principle reversible, as demonstrated with small molecules such as peptides, when the tetrazine containing a sulfide moiety is located between two polymeric chains, this effect is not observed, likely caused by steric effects. However, we demonstrated that this reversibility can be achieved by utilizing oligo PEG with a lower molecular weight.

## Experimental Section

### Materials

All chemicals were used as received, unless otherwise noted. Commercial α-Methoxy-ω-Mercapto PEG2K and PEG5K were purchased from Rapp Polymere GmbH. 7-Methyl-1,5,7,-triazabicyclo[4.4.0]­dec-5-ene (mTBD) (95%), 4-(boc-aminomethy)­benzoic acid (95%), 3-methyl-3-oxetanemethanol (97%), 1-Ethyl-3-(3′-dimethylaminopropyl)­carbodiimide hydrochloride (EDC·HCl) (99%), 4-(dimethylamino)­pyridine (DMAP) (99%), 4-(hydroxymethyl)­benzoic acid (98%), phenyl iodide (III) diacetate (PIDA) (98%), and ter-butylchlorodimethylsilane (98%) were purchased from Fluorochem EU. PEG4-thiol, 1,8-Diazabicyclo[5.4.0]­undec-7ene (DBU) (98%), Imidazole (99%), boron trifluoride etherate (99%), pyridine (99%), trifluoroacetic acid (TFA) (99%), deuterated chloroform (CDCl3, 99.8% with 0.05% v/v TMS), and methyl iodine (99%) were purchased from Sigma-Aldrich. Thiocarbohydrazide (98%) and methyl thiocarbohydrazine were purchased from TCI Europe NV. Anhydrous solvents were obtained by using a solvent purification system from Actu-All Chemicals. l-lactide (Fluorochem EU, 95.0%) was recrystallized once from toluene and once from ethyl acetate prior to use. 5-Acetoxy-*trans*-cyclooctene (TCO-OAc) was donated by the Bonger group (Leiden University).

### Synthesis of MeSTzOH

The synthesis of asymmetrical 1, 2, 4, 5-tetrazine containing a methyl sulfide moiety and a benzyl alcohol was achieved using the protocol by the group of Fox with some modifications.^39^ In brief, 3-methyl-3-oxetanemethanol was reacted with 4-(*tert*-butyl dimethylsilyloxyl)­benzoic acid). The oxetane ester precursor was treated with boron trifluoride and reacted with methyl thiocarbohydrazide to give MeSTzOH. More details for the synthesis and characterization of the methyl thiocarbohydrazide hydrogen iodide salt, 4-(*tert*-butyl dimethylsilyloxyl)­benzoic acid), and the MeSTzOH are reported in the Supporting Information (Figures S8–S14).

### Kinetic Study for the ROP of l-Lactide Utilizing MeSTzOH as the Initiator

In a microwave vial previously dried overnight at 130 °C and cooled down at room temperature under an Argon stream, 100 mg of l-lactide (0.69 mmol, 100 equiv) was placed. The vial was sealed, and the solids were evacuated and purged with vacuum and Argon 3 times. The monomer was solubilized in the selected anhydrous solvent (THF, Toluene, or DCM). A stock solution containing MeSTzOH (0.03 mmol, 5 equiv) and mTBD (0.03 mmol, 5 equiv) in 0.5 mL of the solvent was prepared separately in another microwave vial previously dried overnight in the same conditions. To start the polymerization, 0.1 mL of the stock solution was added to the reaction vial to give a final monomer concentration of 0.2, 0.8, or 1.0 M. Samples at different time points were taken and quenched with a 4-fold excess of benzoic acid dissolved in deuterated chloroform. ^1^H NMR and GPC analysis were performed in order to monitor the monomer conversion, molar mass, and dispersity (Figures S1, S15–S23).

### General Procedure for the ROP of l-Lactide Utilizing MeSTzOH as the Initiator

In a microwave vial previously dried overnight at 130 °C and cooled at room temperature under an argon stream was placed 200 mg of l-lactide (1.39 mmol, 100 equiv). The vial was sealed, evacuated, and purged under vacuum and Argon three times. The monomer was solubilized in 4.52 mL of anhydrous DCM. In another microwave vial previously dried under the same conditions, a stock solution containing 0.02 mmol of MeSTzOH and 0.02 mmol of mTBD in 0.2 mL of anhydrous DCM was prepared. Subsequently, 0.1 mL of the stock solution was added to the reaction vial to start the polymerization. After 60 min, the polymerization was quenched by the addition of 4-fold excess of benzoic acid dissolved in DCM. Excess of solvent was evaporated, and the product was precipitated twice from cold methanol (−20 °C) and centrifuged for 10 min at 4 °C and 4700 rpm. Excess of solvent was evaporated under reduced pressure, and the product was dried overnight under high vacuum to yield a pink powder. ^1^H NMR and GPC analysis was performed in order to obtain the monomer conversion, molar mass, and dispersity. Detailed synthesis and characterization are presented in the Supporting Information (Figures S15 and S23).

### PEG-Tz-PLA by Tetrazine-Thiol Exchange (TeTEx) with α-Methoxy-ω-Mercapto-PEG (PEG-SH)

In a microwave vial, 45 mg of MeSTz-PLA (0.004 mmol, 1 equiv) was solubilized in ACN. In another vial, PEG-SH (*M*_n_ = 2 kDa or 224.32 g mol^–1^) (0.008 mmol, 2 equiv) and TEA (0.008 mmol, 2 equiv) were dissolved in ACN. The solution was transferred to the reaction vial to give a final MeSTz-PLA concentration of 1.0 μmol mL^–1^. The vial was sealed and bubbled with an Argon stream for different reaction times and monitored by GPC. After completion, the polymer was purified by precipitation from cold methanol (−20 °C) and subsequently centrifuged for 10 min at 4 °C and 4700 rpm. The product was transferred to a glass vial by small addition of DCM. Excess of solvent was removed under reduced pressure, and the product was dried overnight under high vacuum to yield a pink solid.

### Reversibility Test of TeTEx by the Addition of Glutathione

In a microwave vial, PEG_n_-Tz-PLA (0.001 mmol, 1 equiv) was solubilized in 0.2 mL of ACN. A solution containing l-glutathione (0.002 mmol, 2 equiv) and triethyl amine in 26.1 μL of ACN was added to the reaction vial. The mixture was vortexed for complete solubilization. The reaction was stirred under constant argon bubbling. After different time points, the mixture was analyzed by GPC.

### TCO Ligation to PEG-Tz-PLA

In a microwave vial covered in aluminum foil, 15 mg of PEG_2kDa_-Tz-PLA (0.001 mmol, 1 equiv) was dissolved in 1.7 mL of DCM. A drop of 5-acetoxy-*trans*-cyclooctene (TCO-OAc) was added, and the reaction was stirred for 20 min. The solution changed from pink to colorless. The product was precipitated once from cold diethyl ether (−20 °C). The polymer was transferred to a glass vial and dried overnight under high vacuum.

## Supplementary Material



## Notes

Experimental procedures, instrumentation, and characterization data including ^1^H NMR and ^13^ C NMR spectra, GPC elugrams, high-resolution mass spectra (HMRS), and FT-IR spectra (PDF)

## References

[1] Ebrahimi F., Ramezani Dana H. (2022). Poly lactic acid (PLA) polymers: from properties to
biomedical applications. Int. J. Polym. Mater.
Polym. Biomater..

[2] Gentile P., Chiono V., Carmagnola I., Hatton P. V. (2014). An overview of poly
(lactic-co-glycolic) acid (PLGA)-based biomaterials for bone tissue
engineering. Int. J. Mol. Sci..

[3] Hussain M., Khan S. M., Shafiq M., Abbas N. (2024). A review on PLA-based
biodegradable materials for biomedical applications. Giant.

[4] Palacio J., Agudelo N. A., Lopez B. L. (2016). PEGylation
of PLA nanoparticles to
improve mucus-penetration and colloidal stability for oral delivery
systems. Curr. Opin. Chem. Eng..

[5] Xiao R. Z., Zeng Z. W., Zhou G. L., Wang J. J., Li F. Z., Wang A. M. (2010). Recent advances in PEG–PLA
block copolymer nanoparticles. Int. J. Nanomed..

[6] Naser A. Z., Deiab I., Darras B. M. (2021). Poly (lactic acid)­(PLA) and polyhydroxyalkanoates
(PHAs), green alternatives to petroleum-based plastics: a review. RSC Adv..

[7] Moins S., Hoyas S., Lemaur V., Orhan B., Delle Chiaie K., Lazzaroni R., Taton D., Dove A. P., Coulembier O. (2020). Stereoselective
ROP of rac-and meso-Lactides Using Achiral TBD as Catalyst. Catalysts.

[8] Masutani, K. , Kimura, Y. Chapter 1. PLA Synthesis. From the Monomer to the Polymer. In Poly(lactic acid) Science and Technology: Processing, Properties, Additives and Applications, A., Jiménez , Peltzer, M. , Ruseckaite, R. , Eds.; The Royal Society of Chemistry, 2014; chapter 1, 1–36, 10.1039/9781782624806-00001.

[9] Danafar H., Rostamizadeh K., Davaran S., Hamidi M. (2014). PLA-PEG-PLA
copolymer-based
polymersomes as nanocarriers for delivery of hydrophilic and hydrophobic
drugs: preparation and evaluation with atorvastatin and lisinopril. Drug Dev. Ind. Pharm..

[10] Yu Y., Storti G., Morbidelli M. (2011). Kinetics of ring-opening polymerization
of l, l-lactide. Ind. Eng. Chem. Res..

[11] Robert J. L., Aubrecht K. B. (2008). Ring-opening polymerization
of lactide to form a biodegradable
polymer. J. Chem. Educ..

[12] Clark L., Deacon G. B., Forsyth C. M., Junk P. C., Mountford P., Townley J. P., Wang J. (2013). Synthesis and structures of calcium
and strontium 2, 4-di-tert-butylphenolates and their reactivity towards
the amine co-initiated ring-opening polymerisation of rac-lactide. Dalton Trans..

[13] Clark L., Cushion M. G., Dyer H. E., Schwarz A. D., Duchateau R., Mountford P. (2010). Dicationic and zwitterionic catalysts for the amine-initiated,
immortal ring-opening polymerisation of rac-lactide: facile synthesis
of amine-terminated, highly heterotactic PLA. Chem. Commun..

[14] Simon L., Goodman J. M. (2007). The mechanism of TBD-catalyzed ring-opening polymerization
of cyclic esters. J. Org. Chem..

[15] Dove A. P. (2012). Organic
catalysis for ring-opening polymerization. ACS
Macro Lett..

[16] Pothupitiya J. U., Dharmaratne N. U., Jouaneh T. M. M., Fastnacht K. V., Coderre D. N., Kiesewetter M. K. (2017). H-Bonding
Organocatalysts for the
Living, Solvent-Free Ring-Opening Polymerization of Lactones: Toward
an All-Lactones, All-Conditions Approach. Macromolecules.

[17] Fasehee H., Dinarvand R., Ghavamzadeh A., Esfandyari-Manesh M., Moradian H., Faghihi S., Ghaffari S. H. (2016). Delivery of disulfiram
into breast cancer cells using folate-receptor-targeted PLGA-PEG nanoparticles:
in vitro and in vivo investigations. J. Nanobiotechnol..

[18] Cai Y., Xu Z., Shuai Q., Zhu F., Xu J., Gao X., Sun X. (2020). Tumor-targeting peptide functionalized PEG-PLA micelles
for efficient
drug delivery. Biomater. Sci..

[19] Liu Y., Xu C., Fan X., Loh X. J., Wu Y.-L., Li Z. (2020). Preparation
of mixed micelles carrying folates and stable radicals through PLA
stereocomplexation for drug delivery. Mater.
Sci. Eng. C.

[20] Pagels R. F., Pinkerton N. M., York A. W., Prud’homme R.
K. (2020). Synthesis
of Heterobifunctional Thiol-poly (lactic acid)-b-poly (ethylene glycol)-hydroxyl
for Nanoparticle Drug Delivery Applications. Macromol. Chem. Phys..

[21] Gavriel K., van Doeselaar D. C., Geers D. W., Neumann K. (2023). Click’n lock:
rapid exchange between unsymmetric tetrazines and thiols for reversible,
chemoselective functionalisation of biomolecules with on-demand bioorthogonal
locking. RSC Chem. Biol..

[22] Ortega-Zamora C., González-Sálamo J., Rivero D. S., Carrillo R., Hernández-Borges J. (2024). Tetrazine-based dynamic covalent
polymers as degradable extraction materials in sample preparation. Anal. Chim. Acta.

[23] Rivero D. S., Paiva-Feener R. E., Santos T., Martín-Encinas E., Carrillo R. (2021). Tetrazine
dynamic covalent polymer networks. Macromolecules.

[24] Van
Den Berg S. A., Zuilhof H., Wennekes T. (2016). Clickable polylactic
acids by fast organocatalytic ring-opening polymerization in continuous
flow. Macromolecules.

[25] Johann K., Svatunek D., Seidl C., Rizzelli S., Bauer T. A., Braun L., Koynov K., Mikula H., Barz M. (2020). Tetrazine-and
trans-cyclooctene-functionalised polypept (o) ides for fast bioorthogonal
tetrazine ligation. Polym. Chem..

[26] Hansell C. F., Espeel P., Stamenovic M. M., Barker I. A., Dove A. P., Du Prez F. E., O’Reilly R. K. (2011). Additive-free
clicking for polymer
functionalization and coupling by tetrazine–norbornene chemistry. J. Am. Chem. Soc..

[27] Mezzasalma L., Dove A. P., Coulembier O. (2017). Organocatalytic ring-opening polymerization
of L-lactide in bulk: A long standing challenge. Eur. Polym. J..

[28] Dechy-Cabaret O., Martin-Vaca B., Bourissou D. (2004). Controlled ring-opening polymerization
of lactide and glycolide. Chem. Rev..

[29] Kamber N. E., Jeong W., Waymouth R. M., Pratt R. C., Lohmeijer B. G., Hedrick J. L. (2007). Organocatalytic
ring-opening polymerization. Chem. Rev..

[30] Kaim W. (2002). The coordination
chemistry of 1, 2, 4, 5-tetrazines. Coord. Chem.
Rev..

[31] Raj K. A., Rao M. R. (2025). Functional 1, 2,
4, 5-Tetrazine Systems for Photocatalysis
and Sensing. Asian J. Org. Chem..

[32] Geers D. W., Gavriel K., Neumann K. (2024). Rapid, traceless and
facile peptide
cyclization enabled by tetrazine-thiol exchange. J. Pept. Sci..

[33] Tallon A.
M., Xu Y., West G. M., Am Ende C. W., Fox J. M. (2023). Thiomethyltetrazines
are reversible covalent cysteine warheads whose dynamic behavior can
be “switched off” via bioorthogonal chemistry inside
live cells. J. Am. Chem. Soc..

[34] Versteegen R. M., Rossin R., ten Hoeve W., Janssen H. M., Robillard M. S. (2013). Click to
release: instantaneous doxorubicin elimination upon tetrazine ligation. Angew. Chem., Int. Ed..

[35] Richter D., Lakis E., Piel J. (2023). Site-specific bioorthogonal protein
labelling by tetrazine ligation using endogenous β-amino acid
dienophiles. Nat. Chem..

[36] Neumann K., Jain S., Geng J., Bradley M. (2016). Nanoparticle “switch-on”
by tetrazine triggering. Chem. Commun..

[37] Rutjes F. P., Bonger K. M., Neumann K. (2024). Bioorthogonal
Chemistry at Radboud
University: Past, Present and Future. Synlett.

[38] Chen W., Wang D., Dai C., Hamelberg D., Wang B. (2012). Clicking 1, 2, 4, 5-tetrazine and
cyclooctynes with tunable reaction
rates. Chem. Commun..

[39] Xie Y., Fang Y., Huang Z., Tallon A. M., Am Ende C. W., Fox J. M. (2020). Divergent Synthesis of Monosubstituted and Unsymmetrical
3, 6-Disubstituted Tetrazines from Carboxylic Ester Precursors. Angew. Chem..

