# Supplementary material for: Multi-postpolymerization Functionalization of PLA Enabled by Sulfide Tetrazines as a Single End-Group Moiety
Source: ACS Polym Au. 2025 Jul 17;5(5):537–44. doi: 10.1021/acspolymersau.5c00045 (PMC12511965; doi:10.1021/acspolymersau.5c00045)
Supplement: Supplementary file 1 [file lg5c00045_si_001.pdf]

# Supporting Information for

## Multi post-polymerization functionalization of PLA enabled by sulfide tetrazines as a single end-group moiety.

**Jesper S. Willems,[1 †] Dulce M. Sánchez-Cerrillo,[1 †] Katerina Gavriel,[1] Kevin Neumann[1*]**

[1]Systems Chemistry Department, Institute for Molecules and Materials, Radboud University Nijmegen.

Heyendaalseweg 135, 6525 AJ Nijmegen, The Netherlands.

*Corresponding author. Email: kevin.neumann@ru.nl; ORCID: 0000-0002-6683-0774

[†]Equal contribution

**Instrumentation**

**Nuclear Magnetic Resonance (NMR)** spectroscopy was performed using a Bruker AVANCE III 400 MHz instrument, equipped with a BBFO probe. A known amount of material, approximately 5-10 mg were resuspended in 600 µL of DMSO-$d_6$ or $CDCl_3$ as NMR solvents. The reported chemical shifts are provided in ppm; residual $^1$H resonance from deuterated solvent is used to reference the $^1$H spectra with the methyl resonance of TMS at 0.0 ppm.

**Gel permeation Chromatography (GPC)** were recorded on a Shimadzu SEC, equipped with a guard column and a PL gel 5 µm mixed D column (Polymer Laboratories). Data was collected by a differential refractive index (RI) and UV ($\lambda$ = 254 nm and $\lambda$ = 280 nm) detection using *N,N*-dimethylacetamide (DMAc) at 50 °C as eluent, at 1.0 mL/min flow rate. The system was calibrated with PS standards.

**Electrospray ionization mass spectrometry (ESI-MS)** was performed on a Single-Quad Thermo instrument equipped with a Thermo Scientific Accucore C18 (2.6 µm, 80 Å, 100 x 3 mm) column using 0.1 % formic acid in ACN and in Mili Q as eluents and differential refractive index (RI) and UV absorbance ($\lambda$ = 254 nm). The mass spectrometer was operating in the positive ion mode.

**High-resoluion mass spectra (HRMS**) was recorded on a JEOL AccuTOF JMS-T100CS

**Matrix-assisted laser desorption/ionization time-of-flight (MALDI-TOF)** mass spectrometry was performed on a Bruker Microflex LRF MALDI-TOF system equipped with a nitrogen laser (337 µm) and operating in reflection mode. Saturating matrix solution was prepared by dissolving *trans*-2-[3-(4-*tert*-Butylphenyl)-2-methyl-2-propenylidene]malonitrile (DCBT) (50 mg/mL) and NaI (150 mg/mL) in THF. 1µl of the matrix solution was loaded on the MALDI plate. The analyte was dissolved in THF and the solution of the analyte and the matrix was mixed in equal ratio. 1.0 µL solution was loaded on the MALDI plate and allowed to dry at room temperature before inserting into the vacuum chamber of the MALDI instrument.

**UV-Vis spectra** was recorded on a UV-2700 UV-Vis spectrophotometer (Sjimadzu Corporation, Japan).

## Synthesis

### Methyl thiocarbohydrazide hydrogen iodide salt

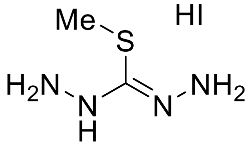


The compound was synthesized as reported previously in literature.[1] In a pre-dried round-bottom flask, 10.0 g of thiocarbohydrazine (94.2 mmol, 1 eq.) and 6.5 mL of methyl iodine (104 mmol, 1.1 eq.) were solubilized in ethanol to give a final concentration of 3 mol $L^{-1}$. The solution was stirred and refluxed at 80 °C under inert atmosphere and left to cool down at room temperature. After completion, the reaction mixture was diluted with 500 mL of n-heptane and stored at -20 °C overnight. The product was collected by filtration and washed with an ethanol:heptane [1:1 v/v] mixture and dried under reduced pressure. The product was obtained as a white solid. Yield: 72%

**$^{1}$H NMR** [ppm] (400 MHz, DMSO-$d_6$): $\delta$ 9.65 (s, 1H), 5.13 (s, 3H), 2.38 (s, 3H).

**ESI-MS**: calculated for $C_2H_9N_4S^+$ $[M+H]^+$: 121.1, found 121.3.

### 4-(Tert-butyl dimethylsilyloxyl)benzoic acid

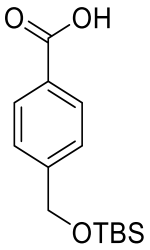


In a pre-dried flask, 8.0 g of 4-(hydroxymethyl)benzoic acid (52.6 mmol, 1.0 eq.), 31.4 g of tert-buthyl chloro dimethylsilane (208 mmol, 4.0 eq.) and 21.5 g of imidazole (315 mmol, 6.0 eq.) were added. Solids were solubilized in anhydrous DMF to give a final concentration of 0.3 mol $L^{-1}$and stirred at room temperature overnight. The reaction was quenched by addition of water, and product was extracted twice with heptane. The organic layer was washed with water (3x), and brine (3x), and subsequently dried over $NaSO_4$. The resulting solution was diluted with water to give a cloudy solution and product was extracted with ethyl acetate. The organic layer was dried over $MgSO_4$. Excess of solvent was removed under reduced pressure.

**$^{1}$H NMR** [ppm] (400 MHz, $CDCl_3$) $\delta$ 8.08 (d, $J$ = 8.2 Hz, 2H), 7.42 (d, $J$ = 8.0 Hz, 2H), 4.81 (s, 2H), 0.96 (s, 9H), 0.12 (s, 6H).

**Oxetane ester precursor**

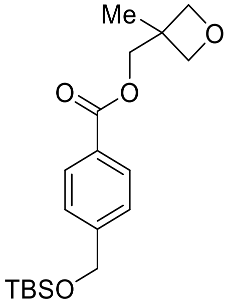


In a flame-dried Schlenk flask, 4.5 mL of 3-methyl-3-oxetanemethanol (45 mmol, 1.1 eq.), 9.5 mg of EDC·HCl (50 mmol, 1.2 eq.), and 0.5 g of DMAP (4.1 mmol, 0.1 eq) were added. Solids were solubilized in anhydrous DCM to give a final concentration of 0.5 mol $L^{-1}$. The reaction mixture was cooled down at 0 °C and a solution containing 11.0 g of 4-(Tert-butyl dimethylsilyloxyl)benzoic acid) (41 mmol, 1.0 eq.) in DCM was added and let stirring for 15 minutes. The reaction mixture was warmed up at room temperature and stirred for 2 days. After completion, mixture was diluted with DCM. Organic layer was washed with sat. aq. $NaHCO_3$ (1x), water (1x), and brine (1x), and subsequently dried over $NaSO_4$ and filtered. The crude was purified by flash chromatography (heptane:ethyl acetate 7:1) to give oxetane ester precursor. Yield: 56%

**$^{1}$H NMR** [ppm] (400 MHz, $CDCl_3$) $\delta$ 8.00 (d, $J$ = 8.4 Hz, 2H), 7.39 (d, $J$ = 8.3 Hz, 2H), 4.77 (s, 2H), 4.62 (d, $J$ = 6.0 Hz, 2H), 4.42 (d, $J$ = 6.0 Hz, 2H), 4.36 (s, 2H), 1.40 (s, 3H), 0.93 (s, 9H), 0.09 (s, 6H).

**$^{13}$C NMR** [ppm] (101 MHz, $CDCl_3$) $\delta$ 166.42, 147.14, 129.65, 128.51, 125.75, 79.58, 68.89, 64.46, 39.33, 25.92, 21.30, 18.37, -5.29.

**HRMS (ESI)**: calculated for $C_{19}H_{30}O_4SiH^+$ $[M+H]^+$ 351.1986, found 351.1994.

**Methyl-sulfide-TzOH**

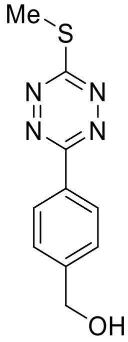

In a flame-dried Schlenk flask, 4.35 g of oxetane ester (12.4 mmol, 1.2 eq.) were solubilized in anhydrous DCM to give a final concentration of 1 mol $L^{-1}$. The solution was cooled down to -12 °C, subsequently 1.54 mg of boron trifluoride etherate (12.5 mmol, 1.2 eq.) was added. The reaction mixture was stirred for 10 min, and 10 mL of anhydrous DCM were added to solvate the mixture again. The reaction continued for additional 4.5 h. Conversion was monitored by TLC of aliquots quenched with trimethylamine. After completion, the mixture was quenched with 2.1 mL of pyridine (26.0 mmol, 2.5 eq.), and subsequently a solution of 2.58 g of methyl thiocarbohydrazide hydrogen iodide salt (10.4 mmol, 1 eq.) in DMF (c = 1 mol $L^{-1}$) was added dropwise to the mixture. The reaction mixture was stirred vigorously under nitrogen flow to evaporate the DCM. The resulting mixture was allowed to stir for 30 minutes at 80 °C under inert conditions. After completion, reaction mixture was cooled down at room temperature, subsequently 3.35 g of PIDA (10.4 mmol, 1.0 eq.) was added to the flaks and the mixture was stirred for additional 30 minutes. After completion, mixture was diluted with DCM and stored overnight at -20 °C. Organic layer was washed with sat. aq. $NaHCO_3$ (1x), water (1x) and brine (1x), and subsequently dried over $MgSO_4$ and filtered. The crude was purified by flash chromatography (heptane:ethyl acetate 9:1 to 1:1). A red powder was obtained and further purified by dissolving in DCM (c = 2.25 mg $mL^{-1}$) and washed with HCl solution 1 mol $L^{-1}$ (1x), water (1x), and brine (1x), and subsequently dried over $MgSO_4$ and concentrated under reduced pressure. The crude was purified by flash chromatography ($CH_2Cl_2$:MeOH 100:0 to 97:3). Product was obtained as a pink powder. Yield: 19.4 %.

**$^1$H NMR** [ppm] (400 MHz, $CDCl_3$) $\delta$ 8.52 (d, $J$ = 8.3 Hz, 2H), 7.57 (d, $J$ = 8.2 Hz, 2H), 4.83 (s, 2H), 2.80 (s, 3H), 1.81 (s, 2H).

**$^{13}$C NMR** [ppm] (101 MHz, $CDCl_3$) $\delta$ 175.40, 162.30, 145.47, 130.97, 127.85, 127.57, 64.93, 13.57.

**HRMS (ESI)**: calculated for $C_{10}H_{10}N_4OSH^+$ $[M+H]^+$ 235.0648, found 235.0653.

**General procedure for the ROP of L-lactide utilizing MeSTzOH as initiator in DCM**

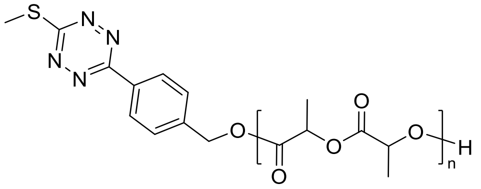

L-lactide (200 mg, 1.39 mmol, 100 eq.) was placed in a microwave vial which previously was dried overnight at 130 °C and cooled down at room temperature under Argon stream. The vial was sealed, evacuated and purged with vacuum and Argon three times. The monomer was solubilized in 4.52 mL of anhydrous DCM. In another microwave vial previously dried under the same conditions, a stock solution containing 0.02 mmol of MeSTzOH and 0.02 mmol of mTBD in 0.2 mL of anhydrous DCM was prepared. Subsequently, 0.1 mL of the stock solution was added to the reaction vial to start the polymerization. After 60 minutes, the polymerization was quenched by the addition of four-fold excess of benzoic acid dissolved in DCM. Excess of solvent was evaporated, and the product was precipitated twice from cold methanol (-20 °C) and centrifuged for 10 minutes at 4 °C and 4700 rpm. Excess of solvent was evaporated under reduced pressure and product was dried overnight under high vacuum to yield a pink powder. $^1$H NMR and GPC analysis was performed in order to obtain the monomer conversion, molar mass and dispersity.

**$^1$H NMR** [ppm] (400 MHz, $CDCl_3$): $\delta$ 5.18 (156 H), 1.60 (460 H).

**GPC** (DMAc, PS calibration) = $M_n$ = 10961 g mol$^{-1}$, $Đ$ = 1.13

**PEG-Tz-PLA by Tetrazine-Thiol exchange (TeTEx) with α-Methoxy-ω-Mercapto-PEG (PEG-SH)**

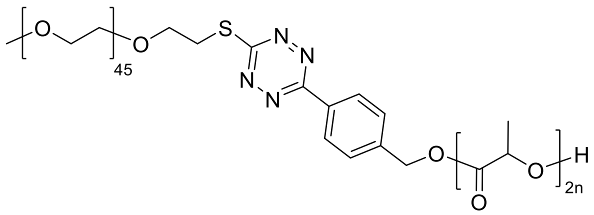

In a microwave vial, MeSTz-PLA (45 mg, 0.004 mmol, 1 eq.) was solubilized in ACN. In another vial, PEG-SH (16 mg, 0.008 mmol, 2 eq.) and TEA (0.008 mmol, 2 eq.) were dissolved in ACN. The solution was transferred to the reaction vial to give a final MeSTz-PLA concentration of 1.0 µmol mL$^{-1}$. The vial was sealed and bubble with Argon stream for 1 hour. After completion the polymer was purified by precipitation from cold methanol (-20 °C) and subsequently centrifuged for 10 min at 4 °C and 4700 rpm. The product was transferred to a glass vial by small addition of DCM. Excess of solvent was removed under reduced pressure and product was dried overnight under high vacuum to yield a pink solid.

$^{1}$**H NMR** [ppm] (400 MHz, $CDCl_3$): $\delta$ 5.18 (156 H), 3.66 (180 H), 1.60 (460 H).

**GPC** (DMAc, PS calibration) = $M_n$ = 12365 g $mol^{-1}$, $Đ$ = 1.11

**Reversibility test of TeTEx by addition of glutathione**

**Using DMSO as organic solvent and 1xPBS**

In a microwave vial PEG-Tz-PLA (3.75 µmol, 1 eq.) and L-glutathione (75 µmol, 20 eq.) were solubilized in 2.63 mL of DMS. 1.12 mL of 1x PBS (pH 6.5) was added to the reaction mixture and vortexed before stirring for 1 h under argon bubbling. After reaction time, the mixture was analyzed by GPC.

**Using Dioxane as organic solvent and 1x PBS**

In a microwave vial PEG-Tz-PLA (5.0 µmol, 1 eq.) and L-glutathione (31 mg, 20 eq.) were solubilized in 3.5 mL of dioxane. 1.5 mL of 1x PBS (pH 6.5) was added to the reaction mixture and vortexed before stirring for 1 h under argon bubbling. After reaction time, the mixture was analyzed by GPC.

**Using ACN as organic solvent and triethyl amine (TEA)**

In a microwave vial, PEG-Tz-PLA (0.001 mmol, 1 eq.) was solubilized in 0.2 mL ACN. A solution containing L-glutathione (0.002 mmol, 2 eq.) and triethyl amine (2eq.) in 26.1 µL ACN was added to the reaction vial. The mixture was vortexed for complete solubilization. The reaction was stirred first for 1 h under constant argon bubbling and then for additional 23 h open to air. After reaction time the mixture was analyzed by GPC.

**Table S1**. Reaction conditions for the synthesis of PEG-Tz-PLA by TeTEx

| Polymer | Tz-PLA | PEG-SH | Solvent | Concentration [µmol $mL^{-1}$] |
|---|---|---|---|---|
| MeSTz-$PLA_{100}$ | 10 µmol | 10 µmol | Dioxane:10xPBS [7]:[3] | 2 |
| MeSTz-$PLA_{20}$ | 10 µmol | 10 µmol | Dioxane:10xPBS [7]:[3] | 2 |
| MeSTz-$PLA_{50}$ | 2 µmol | 2 µmol | Dioxane: 10xPBS [7]:[3] | 1 |
| MeSTz-$PLA_{50}$ | 14 µmol | 10 µmol | DMSO: 10xPBS [7]:[3] | 2 |
| MeSTz-$PLA_{100}$ | 2 µmol | 2 µmol | DMSO: 1xPBS [7]:[3] | 1 |
| MeSTz-$PLA_{100}$ | 2 µmol | 2 µmol | DMSO: 10xPBS [7]:[3] | 1 |
| MeSTz-$PLA_{50}$ | 2 µmol | 2 µmol | ACN | 1 |
| MeSTz-$PLA_{50}$ | 2 µmol | 2 µmol | Dioxane | 1 |

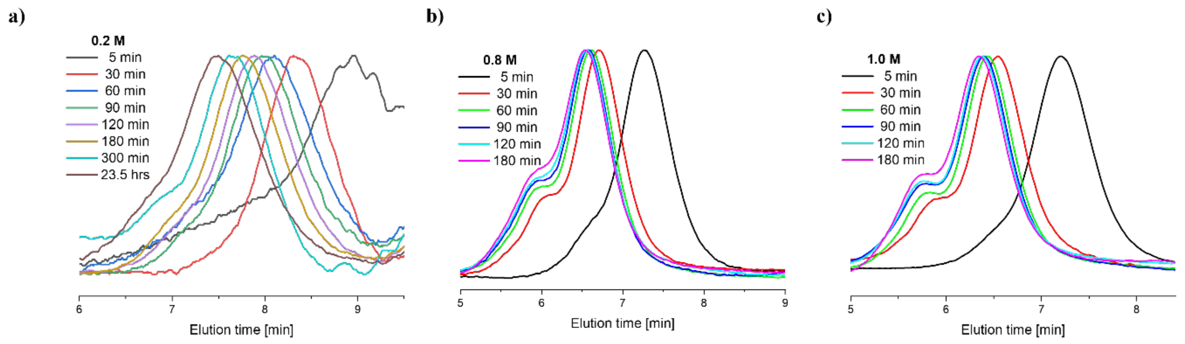


**Figure S1**: Overlay of GPC elugrams for the kinetic study of ROP of L-lactide initiated by MeSTzOH in THF at a) 0.2 M, b) 0.8 M, and c) 1.0 M.

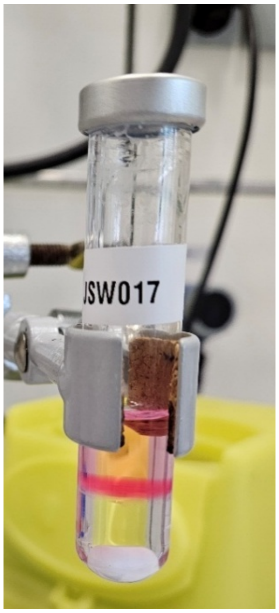


**Figure S2**: Reaction vials for TeTEx in Dioxane and 10xPBS buffer after 1 hour reaction time.

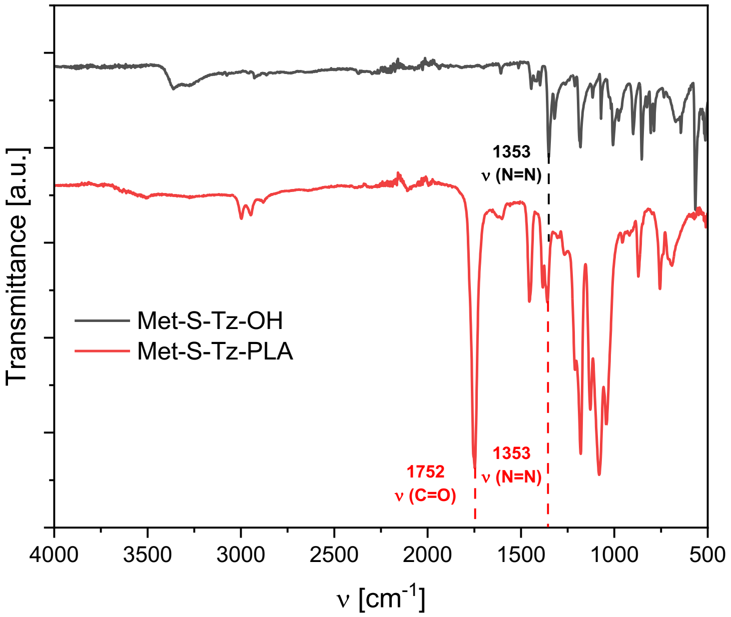


**Figure S3**: FTIR overlay spectra of MeS-Tz-OH and MeS-Tz-PLA.

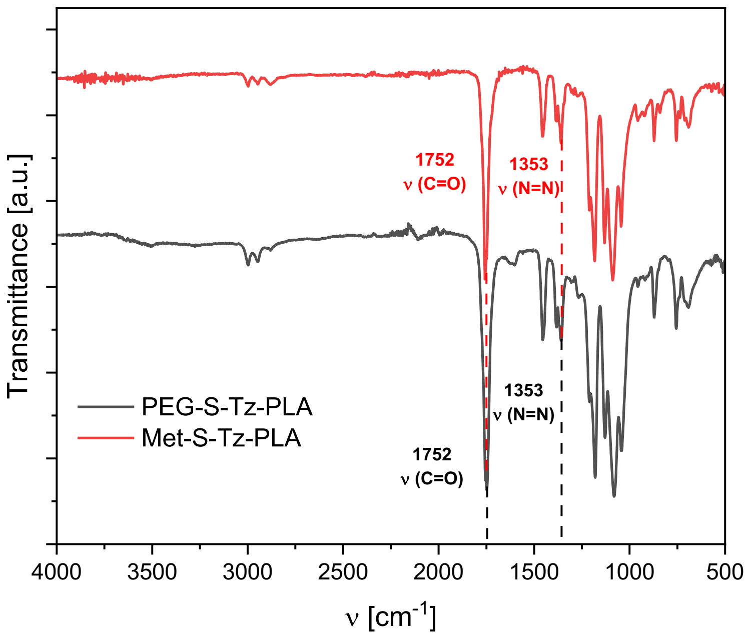


**Figure S4**: FTIR overlay spectra of MeS-Tz-PLA and PEG-S-Tz-PLA

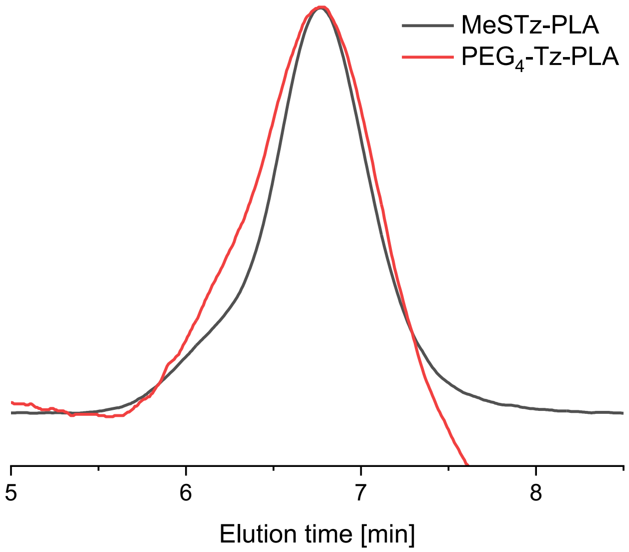


**Figure S5**: Overlay of GPC elugrams (DMAc, RI detection) of MeSTz-PLA and $PEG_4$-Tz-PLA.

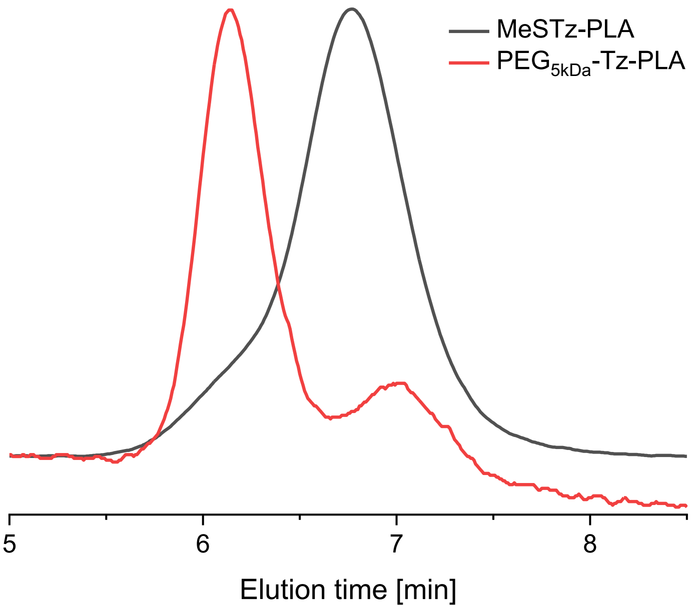


**Figure S6**: Overlay of GPC elugrams (DMAc, RI detection) of MeSTz-PLA and $PEG_{5kDa}$-Tz-PLA.

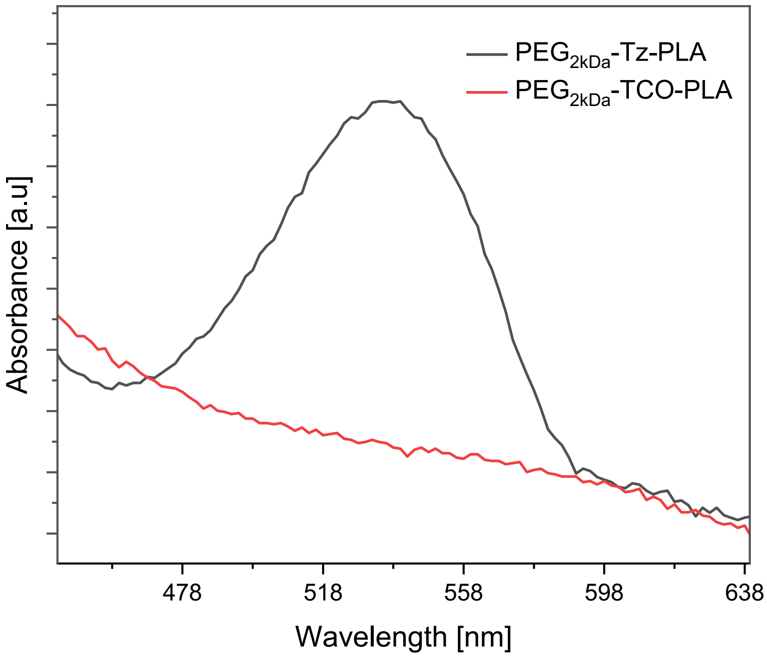


**Figure S7**: UV-Vis overlay spectra of $PEG_{2kDa}$-Tz-PLA and $PEG_{2kDa}$-TCO-PLA

**Characterization Data**

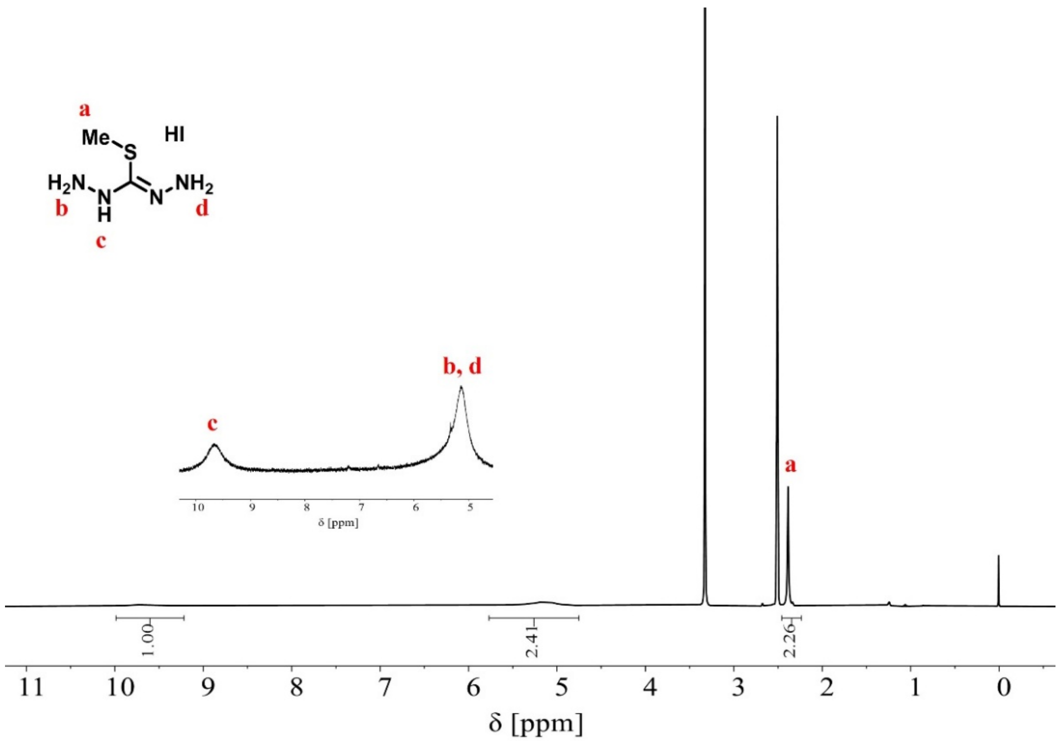


**Figure S8**: $^{1}$H NMR spectrum (400 MHz, DMSO-$d_6$) of Methyl thiocarbohydrazide hydrogen iodide salt.

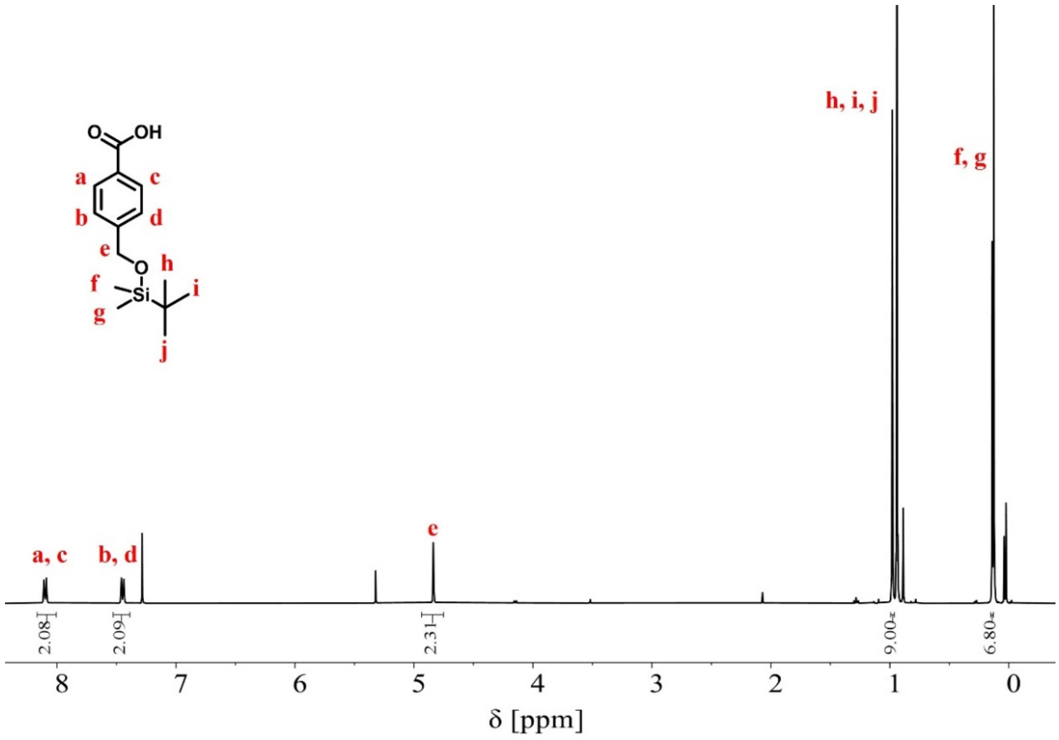


**Figure S9**: $^{1}$H NMR spectrum (400 MHz, $CDCl_3$) of 4-(Tert-butyl dimethylsilyloxyl)benzoic acid.

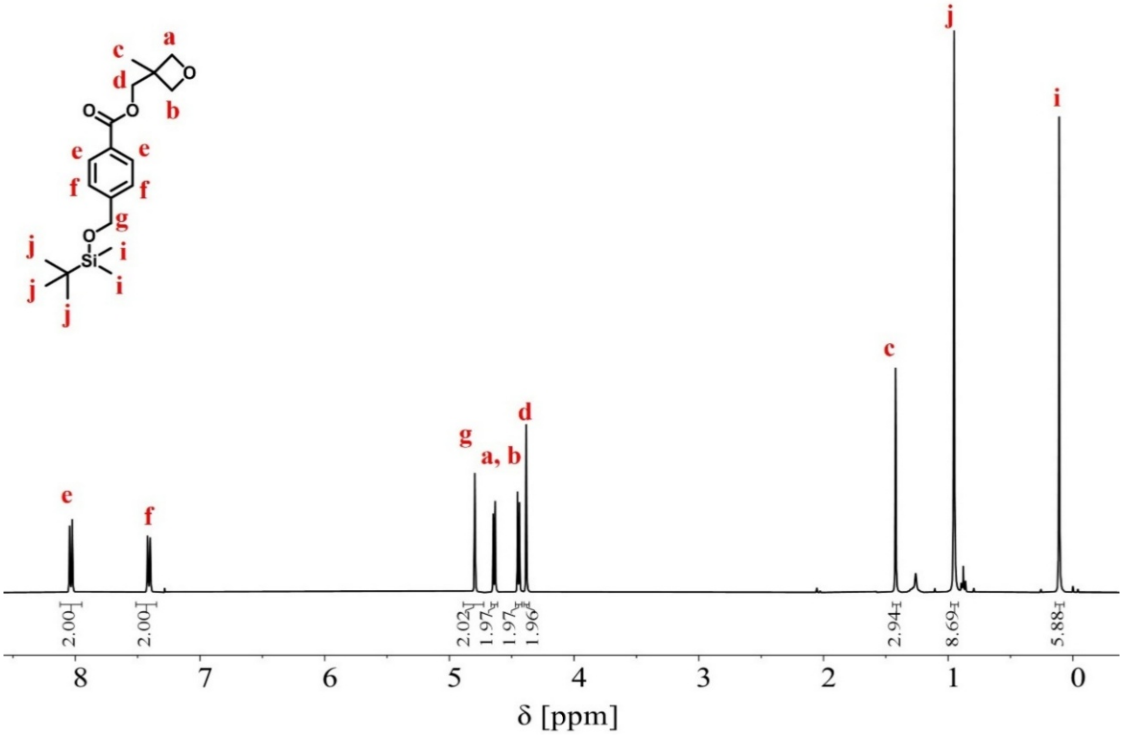


**Figure S10**: $^{1}$H NMR spectrum (400 MHz, $CDCl_3$) of Oxetane ester precursor.

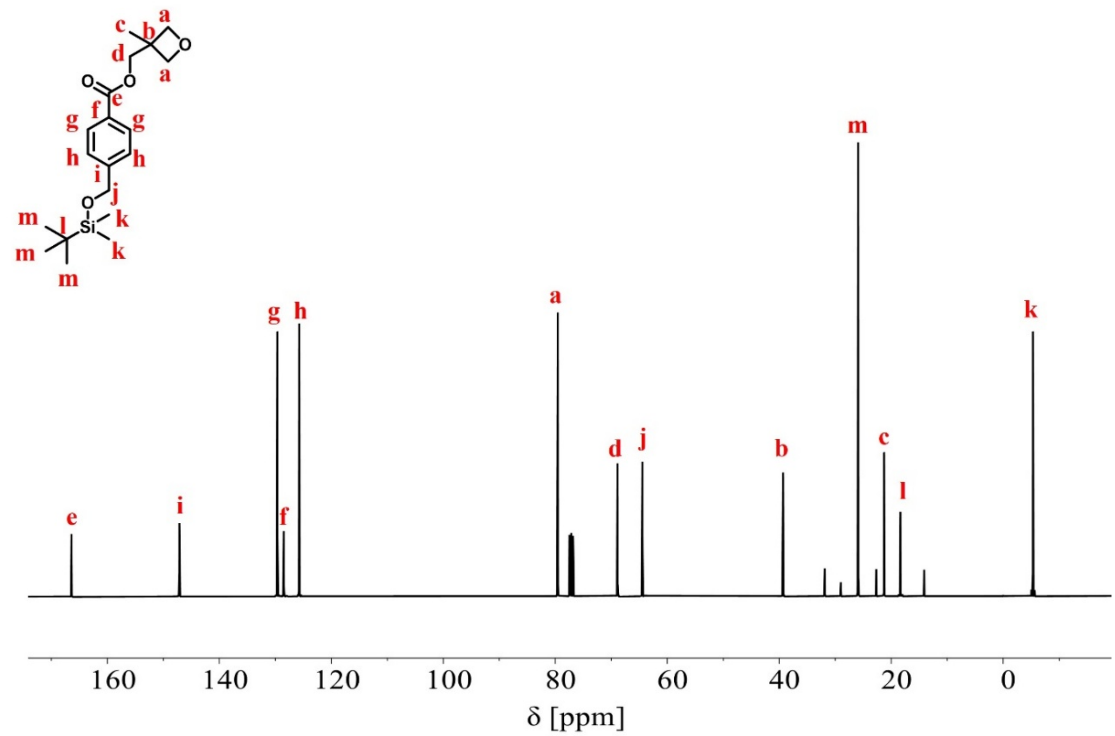


**Figure S11**: $^{13}$C NMR spectrum (101 MHz, $CDCl_3$) of Oxetane ester precursor.

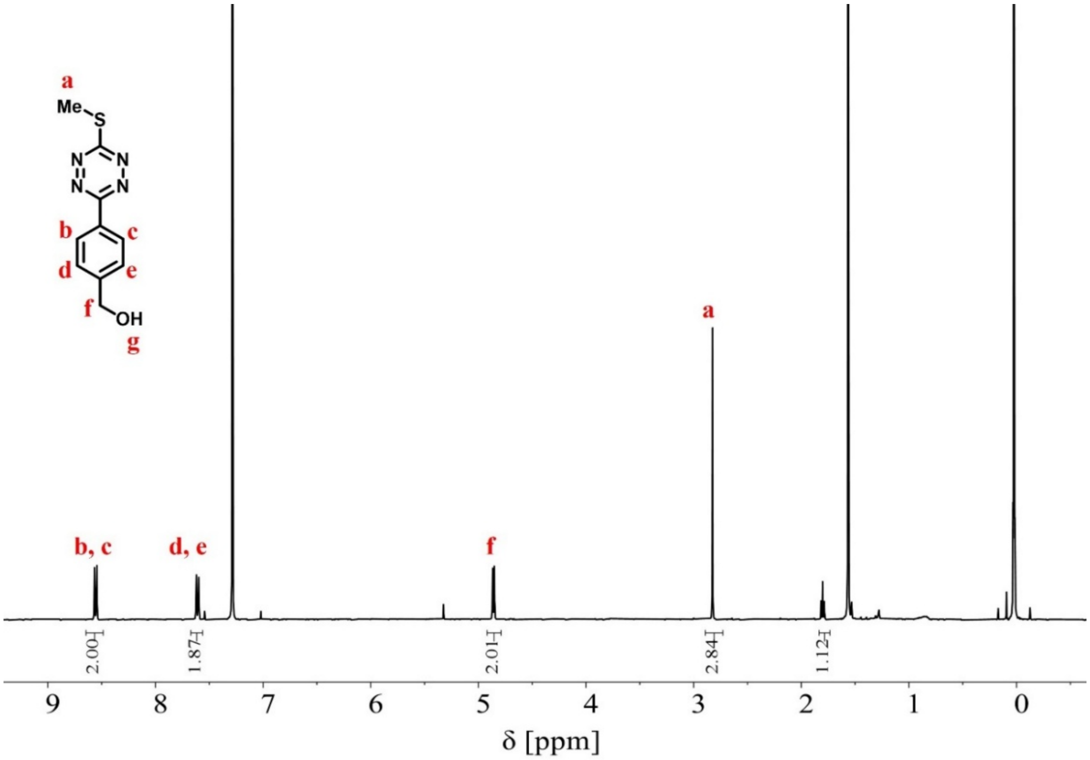


**Figure S12**: $^{1}$H NMR spectrum (400 MHz, $CDCl_3$) of Methyl-sulfide-TzOH.

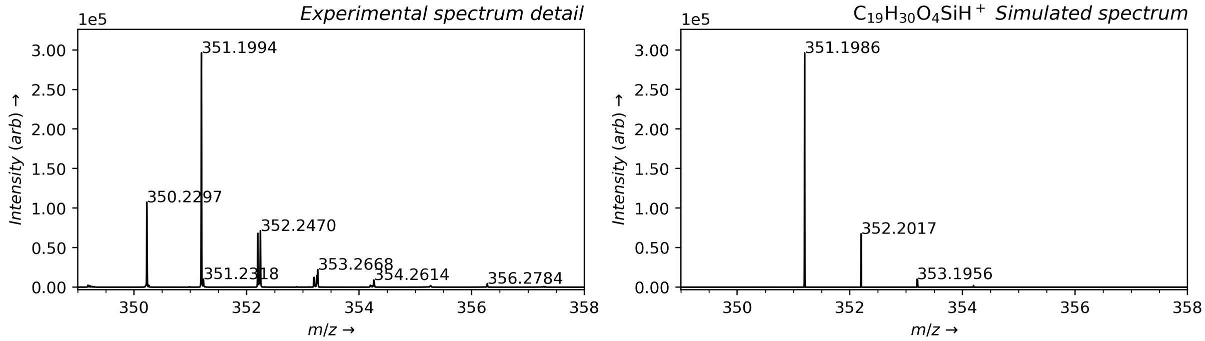


**Figure S13**: HRMS of oxetane ester precursor.

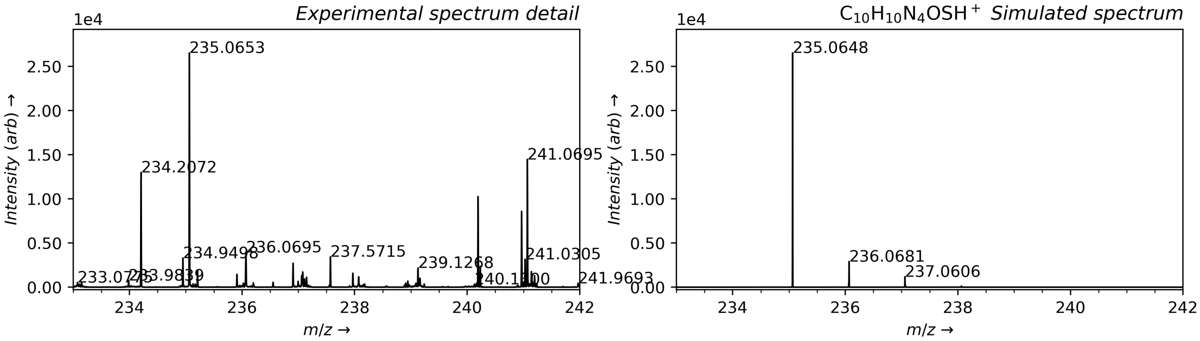


**Figure S14**: HRMS of MeSTzOH.

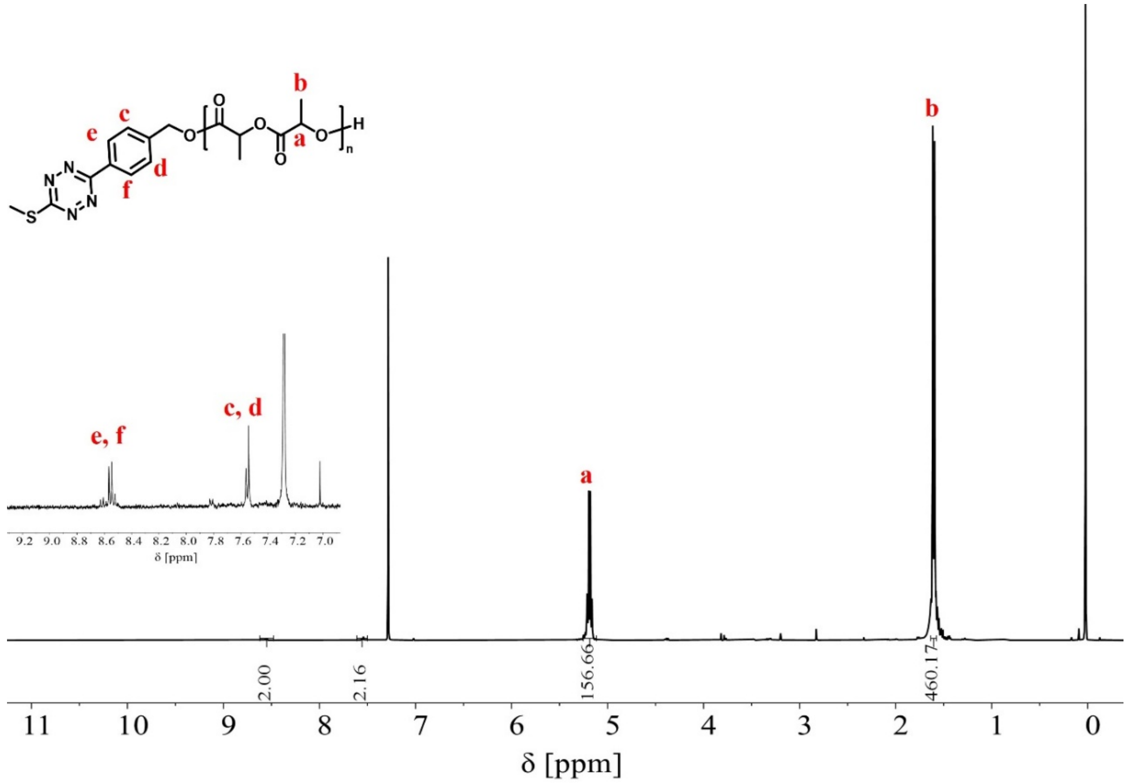


**Figure S15**: $^1$H NMR spectrum (400 MHz, $CDCl_3$) of MeSTz-PLA in DCM 0.3 M.

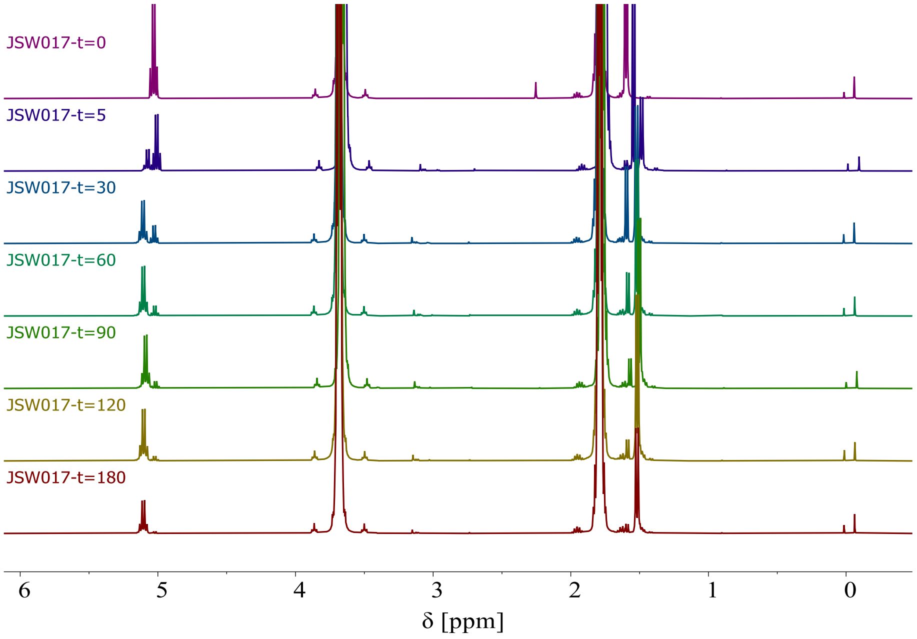


**Figure S16**: $^{1}H$ NMR spectra overlay (400 MHz, $CDCl_3$) for the kinetic study of ROP of L-lactide initiated by MeSTzOH in THF at 1M.

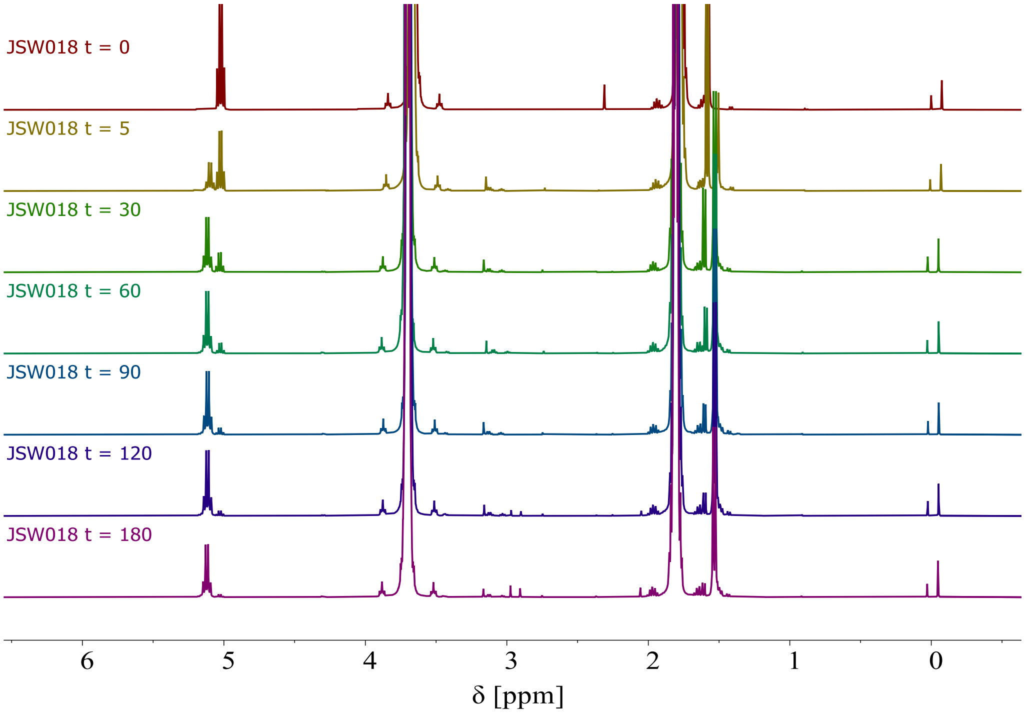


**Figure S17**: $^{1}H$ NMR spectra overlay (400 MHz, $CDCl_3$) for the kinetic study of ROP of L-lactide initiated by MeSTzOH in THF at 0.8 M.

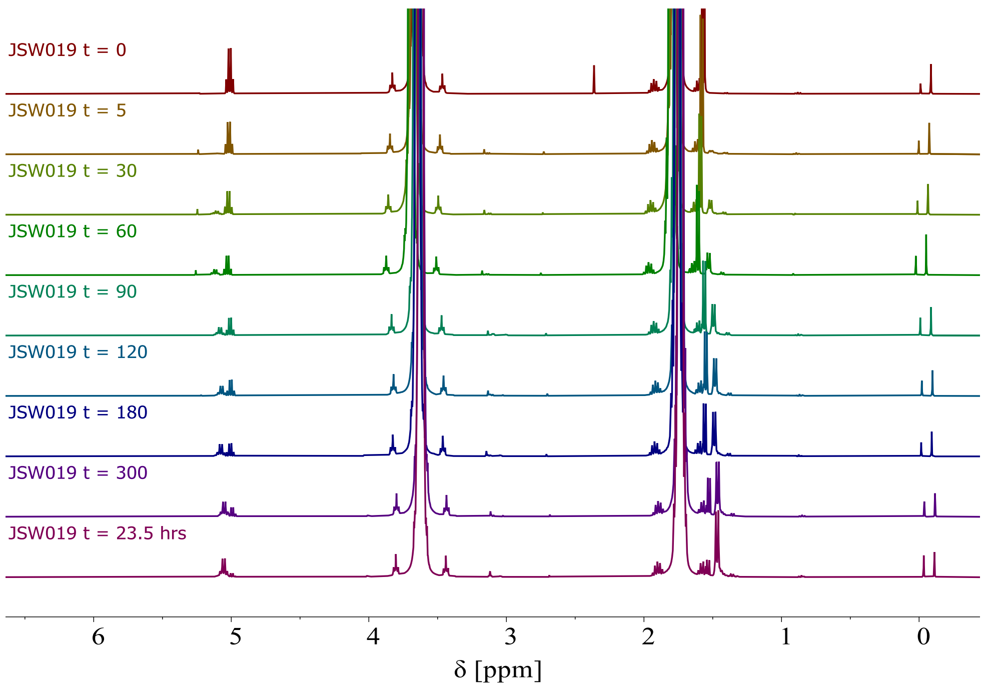


**Figure S18**: $^{1}H$ NMR spectra overlay (400 MHz, $CDCl_3$) for the kinetic study of ROP of L-lactide initiated by MeSTzOH in THF at 0.2 M.

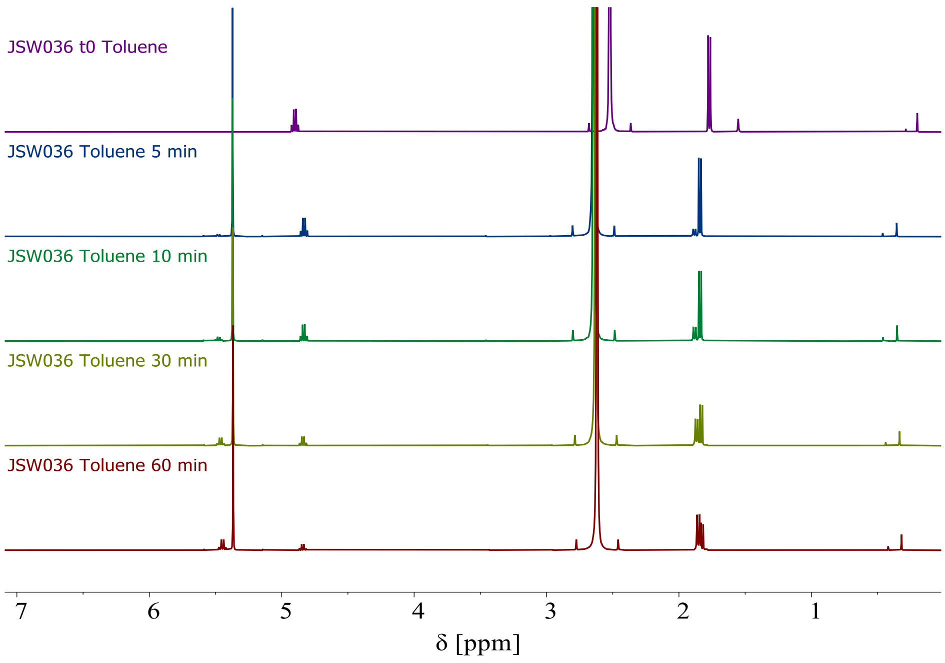


**Figure S19**: $^{1}H$ NMR spectra overlay (400 MHz, $CDCl_3$) for the kinetic study of ROP of L-lactide initiated by MeSTzOH in Toluene at 0.3 M.

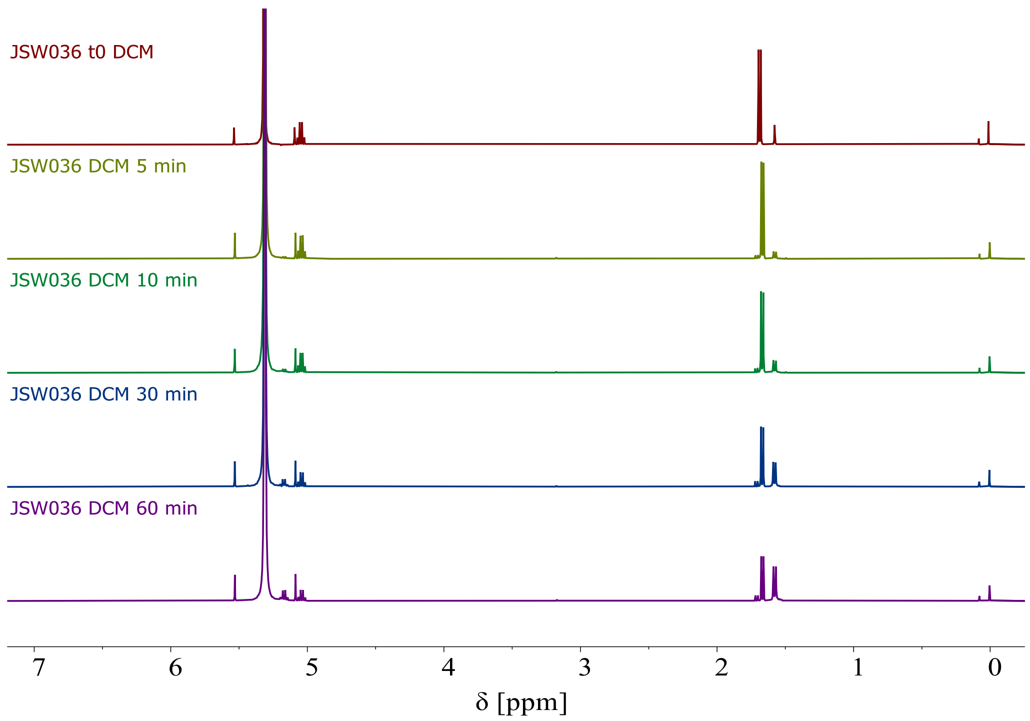


**Figure S20**: $^{1}$H NMR spectra overlay (400 MHz, $CDCl_3$) for the kinetic study of ROP of L-lactide initiated by MeSTzOH in DCM at 0.3 M.

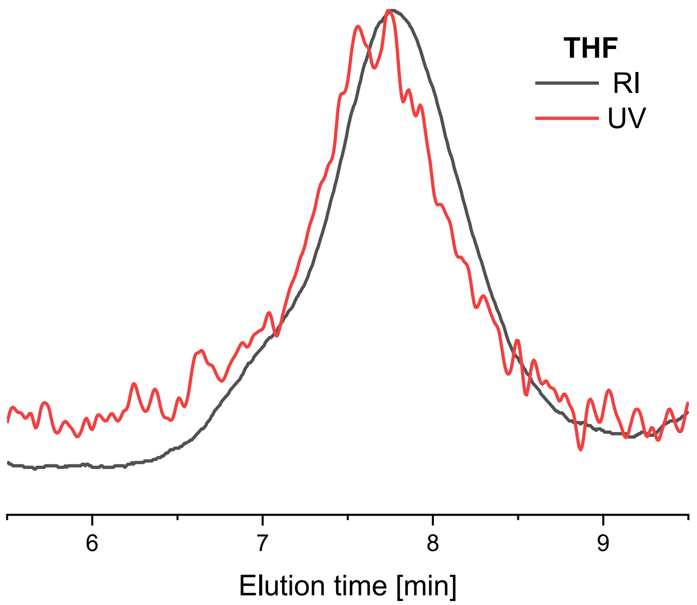


**Figure S21**: Gel permeation chromatography trace of purified PLA utilizing THF as solvent.

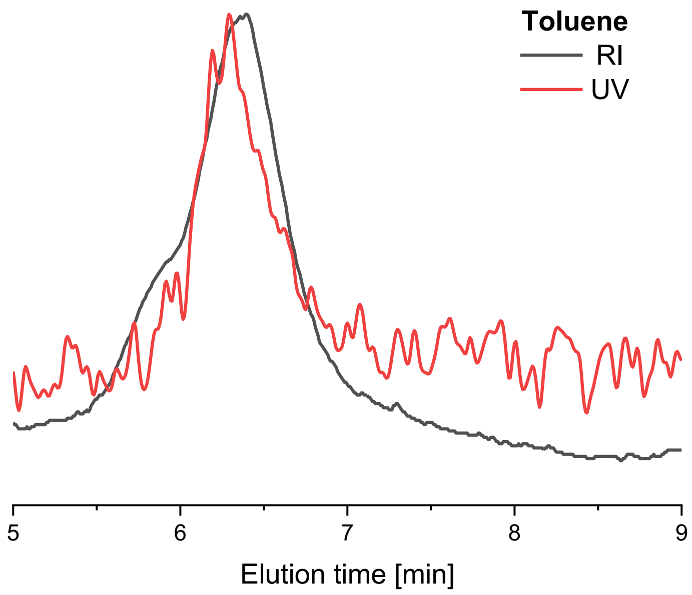


**Figure S22**:  Gel permeation chromatography trace of purified PLA utilizing Toluene as solvent.

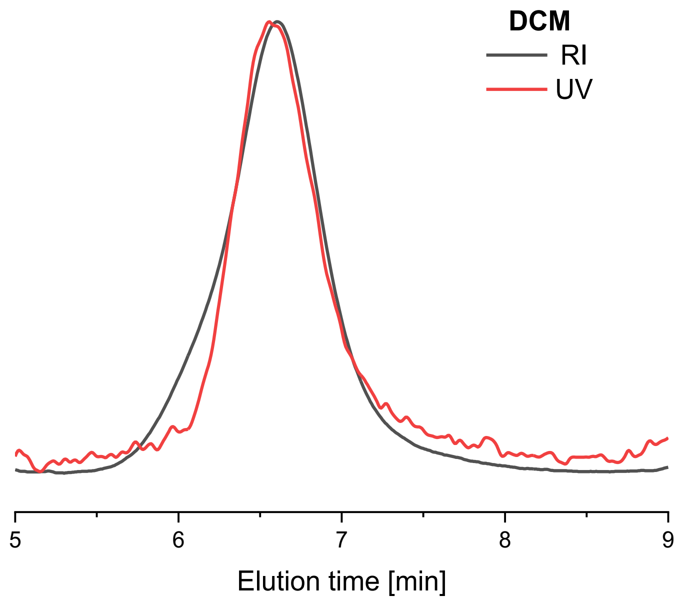


**Figure S23**:  Gel permeation chromatography trace of purified PLA utilizing DCM as solvent.

## References

(1) Xie, Y.; Fang, Y.; Huang, Z.; Tallon, A. M.; Am Ende, C. W.; Fox, J. M. Divergent Synthesis of Monosubstituted and Unsymmetrical 3, 6-Disubstituted Tetrazines from Carboxylic Ester Precursors. *Angewandte Chemie* **2020**, *132* (39), 17115-17121.
